# Dissipation Pathways in a Photosynthetic Complex

**DOI:** 10.1021/acs.jpclett.5c02945

**Published:** 2025-12-12

**Authors:** Ignacio Gustin, Chang Woo Kim, Ignacio Franco

**Affiliations:** † Department of Chemistry, 6927 University of Rochester, Rochester, New York 14627, United States; ‡ Department of Chemistry, 34931 Chonnam National University, Gwangju 61186, South Korea; § The Research Institute for Molecular Science, Chonnam National University, Gwangju 61186, South Korea; ∥ Department of Physics and Astronomy, University of Rochester, Rochester, New York 14627, United States; ⊥ Institute of Optics, University of Rochester, Rochester, New York 14627 United States

## Abstract

Determining how energy flows within and between molecules is crucial for understanding chemical reactions, material properties, and even vital processes such as photosynthesis. While the general principles of energy transfer are well established, elucidating the specific molecular pathways by which energy is funneled remains challenging, as it requires tracking energy flow in complex molecular environments. Here, we demonstrate how photon excitation energy is partially dissipated in the light-harvesting Fenna–Matthews–Olson (FMO) complex, mediating the excitation energy transfer from light-harvesting chlorosomes to the photosynthetic reaction center in green sulfur bacteria. Specifically, we isolate the contribution of the protein and specific vibrational modes of the pigment molecules to the energy dynamics. For this, we introduce an efficient computational implementation of a recently proposed theory of dissipation pathways for open quantum systems, based on second-order perturbation theory in the electronic couplings. Using it and a state-of-the-art FMO model with highly structured and chromophore-specific spectral densities, we demonstrate that energy dissipation is dominated by low-frequency modes (<800 cm^–1^) as their energy range is near-resonance with the energy gaps between electronic states of the pigments. We identify the most important modes for dissipation to be in-plane breathing modes (∼200 cm^–1^) of the bacteriochlorophylls in the complex. Conversely, far-detuned intramolecular vibrations with higher frequencies (>800 cm^–1^) play no role in dissipation. Interestingly, the FMO complex first needs to borrow energy from the environment to release excess photonic energy, indicating that the energy exchange between the system and thermal environment is not strictly unidirectional in time but involves a transient thermally activated step. Beyond their fundamental value, these insights can guide the development of artificial light-harvesting devices and, more broadly, engineer environments for chemical and quantum control tasks.

## Graphical abstract

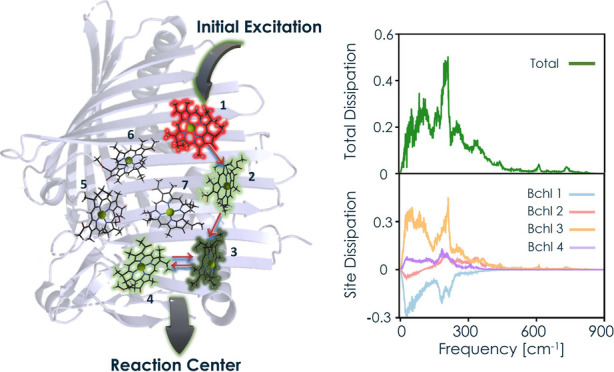

Photosynthesis is a critically important light-induced process that enables plants, algae, and bacteria to convert solar energy into biochemical fuel with remarkable efficiency.^1,2^ This transformation begins with the creation of excitons by solar photons, which travel through a number of pigment molecules until reaching the reaction center, where the energy is transformed into chemical fuel.^3^ During this complex energy-transfer process, excitons dissipate excess energy in the nuclear environment. Although the fundamental principles of energy transfer are well established, mapping the precise molecular pathways of this energy flow remains a formidable task, as it requires tracking energy dynamics in complex molecular environments.

Specifically, it is not understood *how* individual components of the photosynthetic system, including the pigment molecules and their surrounding protein environment, contribute to the overall energy dissipation. Understanding these dissipation pathways is essential for characterizing the energy flow and overall dynamics of photosynthetic processes^3–5^ and is needed to address fundamental questions such as, how do the different components of the pigment molecules contribute to the overall dissipation? Are there specific vibrations that contribute the most to energy dissipation? Is energy transfer a unidirectional process, or do pigment molecules both absorb from and dissipate energy into the environment? Addressing these basic questions requires general strategies to connect the chemical structure to energy transfer dynamics in complex molecular systems.

From an experimental perspective, significant spectroscopic efforts have been made to understand the dynamics of energy transfer within photosynthetic complexes.^6–16^ However, it remains challenging to unravel the intricate energy transfer dynamics in complex chemical environments because the spectral congestion^17,18^ often prevents disentangling contributions by individual components of the pigments and the protein. Progress has been made through two-dimensional electronic spectroscopy (2DES),^19^ which allows tracking exciton dynamics along two frequency axes with femtosecond resolution. Nevertheless, even with these advanced techniques, quantifying the contribution of individual nuclear modes to dissipation remains elusive. This is because the electronic states couple to numerous vibrations, and crucially 2DES projects this high-dimensional vibronic space onto a reduced measurement dimension, thereby limiting the resolution of individual nuclear contributions to energy dissipation.

From a theoretical perspective, the task requires a method to decompose energy dissipation into contributions of the protein and specific vibrational modes of the pigment molecules. The challenge is that this requires quantum dynamical information about this complex chemical environment, and the computational cost of obtaining this information remains beyond the reach of state-of-the-art methods in quantum dynamics. For instance, explicit approaches such as multiconfiguration time-dependent Hartree (MCTDH)^20^ accurately model environmental dynamics through direct wave function propagation, yet they become computationally intractable for macroscopic chemical environments. Trajectory-based mixed quantum-classical methods can also treat complex chemical environments.^21,22^ However, they suffer from artifacts like zero-point energy leakage and negative populations that lead to unphysical dissipation computations.^23,24^ Moreover, their classical treatment of the environment does not correctly capture important quantum processes, such as spontaneous emission. By contrast, quantum master equations and related techniques^25–28^ can treat complex chemical environments implicitly by focusing on the effect in the system’s dynamics. This scalability, however, comes at the cost of discarding information about the environment’s quantum dynamics.

In response to this challenge, we recently developed^29,30^ a general fully quantum theory to quantify and resolve dissipation pathways of quantum systems immersed in highly structured quantum thermal environments, including both harmonic and anharmonic baths. The theory, based on second-order perturbation theory of the off-diagonal system’s couplings, captures the energy dissipated into individual environmental modes while avoiding explicitly propagating the quantum dynamics of the environment, thereby opening a systematic and computationally tractable path to investigate the energy flow between the system and its environment. Here, we introduce an efficient computational implementation (Theoretical Methods) of this theory that now enables access to the dissipation pathways of complex molecular systems while ensuring that the computational cost remains tractable.

We use this strategy to elucidate how photon excitation energy is dissipated in the Fenna–Matthews–Olson (FMO) complex.^31^ This complex mediates the transfer of excitation energy from light-harvesting chlorosomes to the photosynthetic reaction center in green sulfur bacteria.^1,4^ While the structure and exciton dynamics in the FMO have been well studied,^7,8,15,32–54^ the dissipation pathways in the complex have continued to elude both theory and experiments. Specifically, we isolate the contributions of the protein and intramolecular vibrational modes of the pigment molecules to the energy dynamics. We utilize highly structured and pigment-specific spectral densities to characterize the frequencies of protein and intramolecular vibrations, as well as their coupling strengths to electronic excitations in the pigments.^40^ Since these highly structured spectral densities represent high-dimensional environments with 20+ vibrational features per pigment, computing the dissipation pathways is beyond the reach of any other state-of-the-art method in quantum dynamics.

As discussed below, vibrations with frequencies <800 cm^–1^ dominate the energy transfer dynamics, with in-plane breathing modes of the bacteriochlorophylls being the most important ones. While the importance of low-frequency vibrations in the FMO complex has been recognized^55^ our work moves beyond this, elucidating how and why specific modes govern the dissipation dynamics with no a priori assumptions. Interestingly, the simulations also show that the FMO complex dissipates excess photonic energy through a nonmonotonic process, as it initially draws energy from the thermal environment before commencing the overall dissipative process.

The introduced strategy provides a path to disentangle the contribution of individual molecules and nuclear vibrations to the energy dynamics during photosynthetic events.^3–5^ This is important for establishing the connection between the molecular structure and energy dynamics. Beyond its fundamental value, this connection can be helpful for the design of artificial light-harvesting systems,^56–58^ and, more broadly, to engineer environments for chemical and quantum control tasks.^59–64^

Our efforts augment and complement previous efforts to investigate energy pathways in molecular arrays. In particular, there are important strategies to track the energy flow in proteins through vibrational energy transfers using atomistic^65,66^ and coarse-grained^67–72^ classical molecular dynamics simulations. Our approach provides a fully quantum perspective that focuses on the quantification of the dissipation of electronic excitation into nuclear modes. Our efforts also complement strategies^73,74^ developed to isolate the most important coordinate in complex electron transfer processes using electronic structure methods. Such models can be incorporated into our theoretical framework, enabling the analysis of their associated dissipation pathways.

We analyze how energy is dissipated into the thermal environment in the Fenna–Matthews–Olson (FMO) complex, which is essential for efficient light harvesting in green sulfur bacteria such as *Chlorobaculum tepidum* and *Prosthecochloris aestuarii*. The FMO complex acts as a molecular bridge, facilitating the transfer of excitation energy from the chlorosome, where photons are absorbed, to the reaction center, where this energy is converted into chemical fuel. While this transfer is mediated by exciton dynamics within a trimeric FMO complex, research suggests that each exciton’s pathway is confined to a single monomer during transfer,^4^ each housing eight bacteriochlorophyll (Bchl) molecules (see Figure 1a). Therefore, we focus on a single monomer.

**Figure 1:**
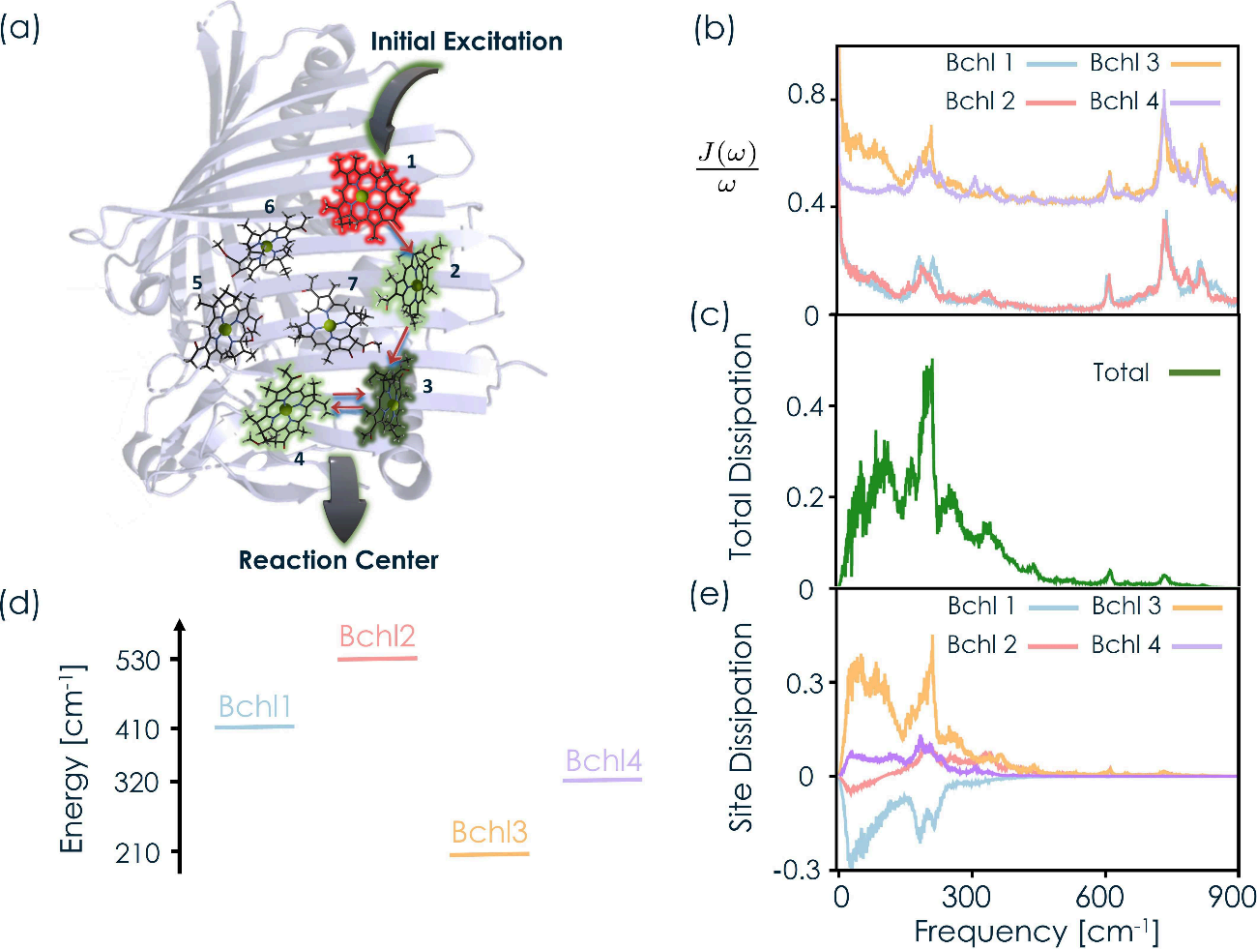
**Dissipation pathways in the FMO complex.** (a) Schematic illustration of the energy dynamics in the FMO complex. The bacteriochlorophyll (Bchl) chromophores are labeled with bold numbers, and the intensity of the green (red) color indicates the amount of energy being dissipated (absorbed). (b) Scaled spectral density *J*(ω)/ω for Bchl1 (light blue), Bchl2 (pink), Bchl3 (orange), and Bchl4 (purple). The baselines for Bchl3 and Bchl4 were vertically shifted for visual clarity. (c) Total dissipation as a function of the environmental frequency. (d) Site energies for Bchl1, Bchl2, Bchl3, and Bchl4. (e) Dissipation decomposition in terms of individual Bchl chromophores.

To describe the energies and couplings of the excited electronic states in the Bchl array, we adopt the Adolphs and Renger^32^ Hamiltonian (see Sec. SI in the Supporting Information). The model includes 7 of the 8 Bchl, as one of them acts as an incoherent energy funnel exclusively connected to Bchl1.^47^ While reported site energies can differ by up to 100 cm^–1^ across studies,^75^ the overall energy landscape and transfer pathways remain consistent across different parameter sets. The Adolphs and Renger parametrization provides a well-established model that captures this consistent landscape and accurately reproduces the optical spectra of the FMO complex.^32^ The interaction between the Bchls and the surrounding environment is described by Bchl-specific spectral densities, computed by Kim and co-workers,^40^ which accurately capture the nuclear environment’s vibrational frequencies (ω) and their coupling strengths with the Bchls electronic excited state with a frequency resolution of 10^–4^ cm^–1^. These spectral densities show good agreement with the available experiments and consist of broad features at frequencies below 200 cm^–1^ that account for protein contributions and a series of sharp peaks to describe the influence of intramolecular vibrational modes. In Figure 1b, we detail a segment of the spectral density scaled by the environment frequency, *J*(ω)/ω, for the chromophores that are the most important in the process of the absorption/dissipation of energy. The remaining spectral densities are presented in Figure S1. The reliability of our spectral densities, particularly in the challenging low-frequency region, is ensured by the extensive 100 ns MD trajectory employed in its construction. This approach provides a high frequency resolution of 3 × 10^–4^ cm^–1^.^40^

Our analysis is based on a recently proposed fully quantum theory of dissipation pathways^29,76^ (see Theoretical Methods for details), which is used to quantify the amount of energy channeled from the Bchls in the FMO into the complex’s vibrational modes, including both protein and intramolecular contributions. The applicability of our theory, which assumes weak electronic coupling, to compute dissipation pathways in the FMO complex is validated against the numerically exact hierarchical equation of motion (HEOM)^77–79^ using illustrative models (see Secs. SII–SIII in the Supporting Information), showing semiquantitative agreement. We note that direct HEOM calculations of dissipation pathways incorporating the highly structured spectral densities of the FMO complex are currently computationally prohibitive. Thus, our benchmark, using representative models that capture the essential features of the FMO, represents state of the art.

Figure 1 shows the contributions of specific environmental modes at frequency ω to the overall asymptotic energy absorption/dissipation in the overall complex and in each chromophore when the initial excitation is placed in Bchl1, the site closest to the chlorosome.^80^ This electronic excitation moves through the complex until it reaches Bchl3 and Bchl4, the chromophores closest to the reaction center.^32^ In Figure 1a, chromophores are labeled with bold numbers, and the intensity of the green (or red) color indicates the relative amount of energy dissipated (or absorbed) at each chromophore in the overall process.

Figure 1c shows the overall frequency-resolved dissipation profile, which reveals that the dissipation of energy is driven by the low-frequency modes (<800 cm^–1^). This is because their energy is near-resonant with the energy gaps between excited electronic states in the complex (see Figure 1d). In particular, we identify environmental modes around 200 cm^–1^ as the most important modes for dissipation in the FMO complex, giving the most important contribution in Figure 1c. Remarkably, despite being present in the spectral densities^40^ (see Figure S1), high-frequency vibrational modes (>800 cm^–1^) do not contribute to the energy dissipation in this complex as their energy is far detuned from the energy gaps between excited electronic states. This is consistent with previous analyses in the FMO complex^42–46,81^ that showed that high-frequency components are not necessary to capture exciton dynamics accurately.

In Figure 1e, we decompose the overall dissipation into the contribution of the nuclear modes coupled to the most relevant Bchls (1–4). Interestingly, Bchl1 (light blue) has a “negative” dissipation, which means that it absorbs energy from the environment. By contrast, Bchl2 (pink), Bchl3 (orange), and Bchl4 (purple) release energy into the environment, with Bchl3 making the most significant contribution. The remaining Bchls are not shown, as they have a minor impact on the exciton population and energy dissipation dynamics.

To elucidate the vibrational modes around 200 cm^–1^ that predominantly contribute to dissipation in the FMO complex across all chromophores, we developed a computational strategy to extract the representative vibrational motion from the molecular dynamics trajectories used for constructing the spectral densities (see Sec. SIV in the Supporting Information for details). Figure 2 shows the isolated vibrational mode around 200 cm^–1^ for Bchl3, displaying only the Bchl core for enhanced clarity. Interestingly, this vibrational mode, the most responsible for dissipation, exhibits a breathing motion pattern and is confined to the plane with no out-of-plane components. We do not show other chromophores, as the modes around 200 cm^–1^ exhibit remarkable similarity across all chromophores, including Bchl1, which absorbs energy from the environment. A set of animations showing the vibrational mode for every Bchl around 200 cm^–1^ is available in the Supporting Information. Importantly, the relevance of this vibrational frequency around 200 cm^–1^ has been previously noted in 2DES experiments in the FMO complex where including this vibrational mode in the spectral density was essential for obtaining energy transfer rates that align with experimental observations.^15^ Furthermore, theoretical investigations^55^ have also found it necessary to include this mode on an ad hoc basis to reproduce experimental results, reinforcing the consensus of its critical role. By contrast, our work does not presuppose its importance. Instead, the dominant role of the 200 cm^–1^ vibration is a direct outcome of the system’s dynamics, providing a first-principles validation of its function and revealing the underlying molecular mechanism driving the dissipation.

**Figure 2:**
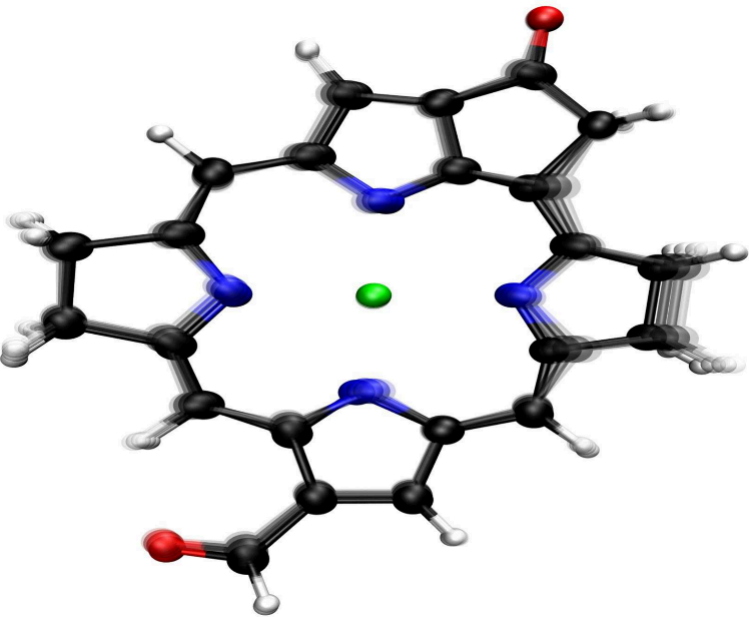
**Vibrational mode driving energy dissipation in the FMO complex.** The figure shows the vibrational mode around 200 cm^–1^ in Bchl3, which is crucial for energy dissipation. This mode is remarkably similar across all chromophores (see animations in the Supporting Information). The mode was extracted from molecular dynamics simulations as detailed in the Supporting Information.

To ensure that these findings are not dependent on the initial excitation site, we conducted a simulation identical with the excitation initiated on Bchl6. These calculations, which are detailed in Sec. SV in the Supporting Information, yield a consistent picture of the energy dissipation dynamics. The process remains dominated by low-frequency nuclear modes (<800 cm^–1^), with the vibrational mode near 200 cm^–1^ consistently emerging as the most significant contributor to energy relaxation. This confirms that the identified dissipation channel is a robust feature of the system’s dynamics.

We now go beyond the overall dissipation and scrutinize the role of each Bchl during the energy transfer dynamics. Figure 3 shows the cumulative dissipation over time for the four most significant chromophores and the overall complex. Figure 3a shows that, initially, the dissipation is negative, indicating that the system is absorbing energy from the environment to facilitate uphill energy transfer from Bchl1 to Bchl2 (see the energy level diagram in Figure 3d). Interestingly, both Bchl1 and Bchl2 collectively absorb energy during this phase. This initial energy absorption is consistent with the uphill energy transfer signals involving Bchl1 and Bchl2 observed experimentally in two-dimensional electronic spectroscopy (2DES) experiments measured at 40 fs.^82^

**Figure 3:**
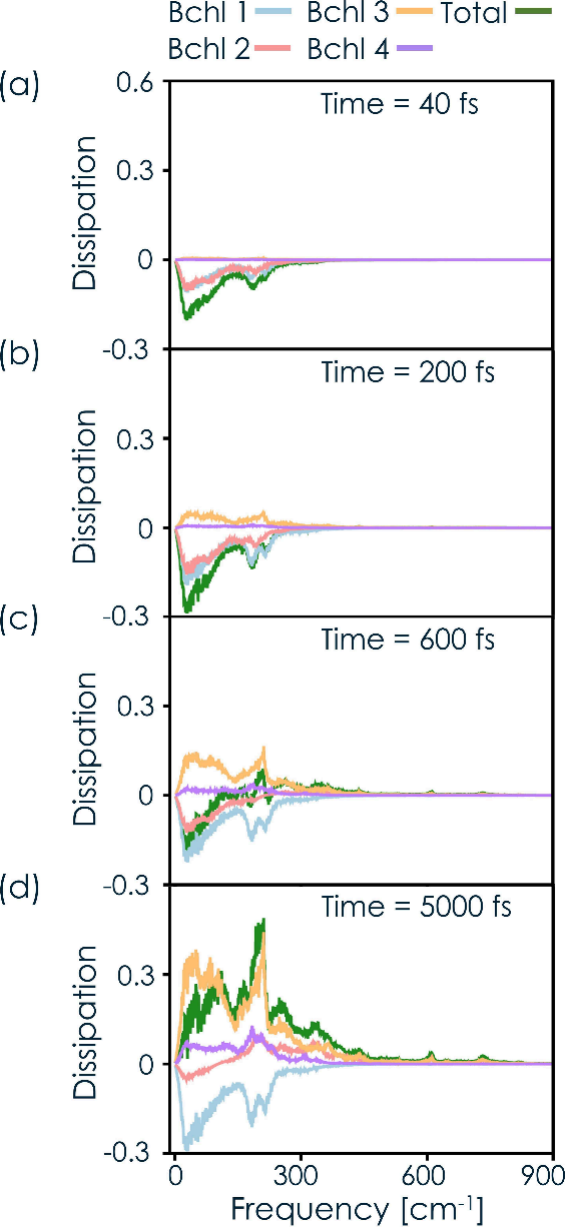
**Time-dependent energy dissipation in the FMO complex.** The panels show the cumulative dissipation by environmental modes at different frequencies at selected times during the dynamics: (a) 40 fs, (b) 200, and (c) 600 fs and the overall dissipation at (d) 5000 fs. The colored lines quantify the contributions of nuclear modes belonging to Bchl1 (light blue), Bchl2 (pink), Bchl3 (orange), and Bchl4 (purple). The green line quantifies the total dissipation. Note that negative values of the dissipation indicate energy absorption.

At 200 fs, as shown in Figure 3b, Bchl3 plays a significant role in overall dissipation due to increased population transfer to this chromophore. By 600 fs, Figure 3c, Bchl1 continues to absorb energy from the environment, while Bchl3 maintains its role in dissipating energy. Meanwhile, Bchl2 reverses its initial energy directionality to dissipate energy into the environment. This change in Bchl2 from absorption to dissipation, along with the ongoing energy dissipation of Bchl3, changes the overall energy profile of the system toward net dissipation into the environment. By 5000 fs, the system reaches a steady state, and it is clear that the overall energy is dissipated into the environment across all frequencies. Interestingly, Bchl1 consistently absorbs energy throughout the process.

This analysis shows that the energy flow within the FMO complex is not unidirectional. Some chromophores absorb energy from or dissipate energy into the environment throughout the dynamics, while others can absorb or dissipate energy on different time scales. Furthermore, the analysis shows that not all vibrational modes are equally important for energy transfer dynamics in the FMO complex. In fact, we identified the in-plane breathing modes around 200 cm^–1^ as the most important for energy transfer.

In conclusion, for the first time, we have successfully isolated the dissipation pathways in the Fenna–Matthews–Olson complex by quantifying contributions by individual environmental components to the energy dynamics. This was achieved through an efficient computational implementation of our recently developed quantum theory of dissipation pathways^29,76^ combined with a state-of-the-art FMO model incorporating highly structured and Bchl-specific spectral densities.

Our analysis revealed that energy dissipation in the FMO complex is predominantly driven by low-frequency vibrational modes (<800 cm^–1^), with in-plane breathing modes around 200 cm^–1^ being the most dominant. High-frequency modes (>800 cm^–1^) play a negligible role despite their presence in the spectral densities as they are far detuned from the excited state electronic energy gaps in the FMO complex. Furthermore, we demonstrated that energy flow within the FMO complex is nonmonotonic, as it involves a transient back-and-forth energy exchange, reflecting a thermally activated step prior to overall dissipation. Specifically, to release excess photonic energy, the FMO complex must first absorb energy from the environment. We identify Bchl1, closest to the chlorosome, as the primary mediator of initial energy absorption and Bchl3, closest to the reaction center, as the primary mediator of energy dissipation.

The ability to precisely map dissipation pathways in complex chemical systems, as demonstrated in this study of the FMO complex, opens new avenues for investigating the role of the environment in energy transfer dynamics. We envision future applications of this methodology extending beyond the FMO complex to other light-harvesting systems, including, but not limited to, light-harvesting complex II (LH2),^3^ phycobiliprotein PC645,^83^ cyanobacterial light-harvesting proteins,^84^ and other molecular systems.^85–89^ Furthermore, our strategy can aid in guiding emerging efforts to develop more efficient artificial light-harvesting systems^90^ by identifying the key vibrational modes and pigment molecules involved in energy transfer in natural light-harvesting systems and, more broadly, advance efforts to control quantum systems via environment engineering.

## Theoretical Methods

In ref 29 we developed MQME-D, a general framework for constructing practical and accurate schemes to isolate dissipation pathways in open quantum systems targeting Markovian quantum master equations (MQME). The formulation incorporates a specific bath degree of freedom into the subsystem component and calculates the change in its energy with the Nakajima-Zwanzig projection operator technique. By perturbatively expanding the Liouville–von Neumann equation, the MQME-D elucidates the rate of dissipation expressed by using traces of operator products, which can be applied for general types of bath and subsystem-bath interaction. The MQME-D also rigorously satisfies thermodynamic principles, such as energy conservation and detailed balance.

Here, we outline the main ideas of our quantum theory of dissipation pathways based on Fermi’s golden rule Markovian quantum master equations describing the open quantum dynamics. We focus on the case when the environment consists of a local set of effective harmonic modes, as is the case with photosynthetic complexes. However, the approach can be applied to any harmonic or anharmonic environment, provided that it can be modeled with independent environment degrees of freedom.^29,30^

We consider an array of *N* chromophores that are coupled to one another and to local thermal environments. The state |*A*⟩ describes a physical situation in which the chromophore *A* is in the excited electronic state, while the other chromophores in the complex are in the electronic ground state. The population dynamics of chromophore *A* is given by

1 $${Ṗ}_{A}\left(t\right)=\underset{B\neq A}{\sum }\left[-K_{BA}P_{A}\left(t\right)+K_{AB}P_{B}\left(t\right)\right]$$

where *K*_*BA*_ is the rate constant of excitation transfer from |*A*⟩ to |*B*⟩ given by

2 $$K_{BA}=\frac{2|V_{AB}{|}^{2}}{\hbar^{2}}R\int_{0}^{\infty }\exp\left[-\frac{it^{\prime }}{\hbar }\left(E_{B}-E_{A}+\Lambda_{A}+\Lambda_{B}\right)\right]\times \exp\left[-g_{A}\left(t^{\prime }\right)-g_{B}\left(t^{\prime }\right)\right]dt^{\prime }$$

Here, *E*_*A*_ and *E*_*B*_ represent the electronic energy gaps from the ground to the excited state at chromophores *A* and *B*, respectively, and *V*_*AB*_ represents the electronic coupling between these chromophores. The total electron-vibrational coupling at a given chromophore *A* is measured by the reorganization energy Λ_*A*_. The reorganization energy is related to the environment spectral density by

3 $$\Lambda_{A}=\int_{0}^{\infty }\frac{J_{A}\left(\omega \right)}{\omega }d\omega$$

The line-broadening function of chromophore *A* is determined by

4 $$g_{A}\left(t\right)=\frac{1}{\hbar }\int_{0}^{\infty }J_{A}\left(\omega \right)\left[\coth\left(\frac{\beta \hbar \omega }{2}\right)\frac{1-\cos\left(\omega t\right)}{\omega^{2}}+i\frac{\sin\left(\omega t\right)-\omega t}{\omega^{2}}\right]$$

where β = 1/*k*_B_ *T* is the inverse thermal energy.

Within this framework, the dissipation rate *Ė*_*Aj*_(*t*) in the *j*-th vibrational mode belonging to the chromophore *A* can be evaluated as

5 $${Ė}_{Aj}\left(t\right)=\underset{B\neq A}{\sum }\left[K_{BA}^{Aj}P_{A}\left(t\right)+K_{AB}^{Aj}P_{B}\left(t\right)\right]$$

where the dissipation rate constants $\left\{K_{BA}^{Aj}\right\}$ are computed as

6 $$K_{BA}^{Aj}=\frac{2|V_{AB}{|}^{2}}{\hbar^{2}}\lambda_{A}^{j}I_{BA}\left(\omega_{Aj}\right)$$

Here, λ_*A*_^*j*^ is the discrete reorganization energy of the *j*-th vibration that belongs to |*A*⟩. If we sum over all the vibrations at chromophore *A* we obtain the total reorganization energy Λ_*A*_ = ∑_*j*_λ_*A*_^*j*^. In turn, $I_{\mathrm{BA}}\left(\omega \right)$ is the “dissipative potential” defined as

7 $$I_{BA}\left(\omega \right)=R\int_{0}^{\infty }\exp\left[-\frac{it^{\prime }}{\hbar }\left(E_{B}-E_{A}+\Lambda_{A}+\Lambda_{B}\right)\right]\times \exp\left[-g_{A}\left(t^{\prime }\right)-g_{B}\left(t^{\prime }\right)\right]\times \left[\cos\left(\omega t^{\prime }\right)-i\coth\left(\frac{\beta \hbar \omega }{2}\right)\sin\left(\omega t^{\prime }\right)\right]dt^{\prime }$$

which quantifies the ability of the *j*-th vibration to induce dissipation per unit of reorganization energy at chromophore *A*. To obtain an expression for the rate of dissipation at chromophore *A* as a function of frequency at a certain time, $D_{A}\left(\omega ,t\right)$ , we replace $K_{BA}^{Aj}$ and $K_{AB}^{Aj}$ with the “dissipative spectral densities” $J_{BA}^{A}\left(\omega \right)$ and $J_{AB}^{A}\left(\omega \right)$

8 JBAA(ω)=2|VAB|2ℏ2JA(ω)ωIBA(ω)JABA(ω)=2|VAB|2ℏ2JA(ω)ωIAB(ω)

where we have used the property ∑_*j*_λ_*A*_^*j*^ = ∫_0_^∞^dω *J*_*A*_(ω)/ω. This replacement yields the dissipation function

9 $$D_{A}\left(\omega ,t\right)=\underset{B\neq A}{\sum }\left[J_{BA}^{A}\left(\omega \right)P_{A}\left(t\right)+J_{AB}^{A}\left(\omega \right)P_{B}\left(t\right)\right]$$

This equation enables us to compute the dissipation of chromophore *A* as a function of time and environment frequency. Importantly, all the terms in eq 9 can be obtained without explicitly accessing the dynamics of the vibrational environment, keeping the computational cost tractable.

The accumulated chromophore dissipation at a given time, $E_{A}\left(\omega ,t\right)$ , can then be obtained as

10 $$E_{A}\left(\omega ,t\right)=\int_{0}^{t}D_{A}\left(\omega ,t^{\prime }\right)dt^{\prime }$$

In turn, the total time-dependent dissipation can be obtained as

11 $$E_{\mathrm{Tot}}\left(\omega ,t\right)=\overset{N}{\underset{A=1}{\sum }}E_{A}\left(\omega ,t\right)$$

where *N* is the number of chromophores.

### Non-Markovian Effects

Equation 1 is a Markovian quantum master equation which assumes that the dynamics of the environment are fast compared to the system’s dynamics. In our simulations, we account for non-Markovian effects that go beyond this treatment by using a time scale separation method^91,92^ to split the spectral density, *J*(ω), into “slow” and “fast” components, denoted as *J*_slow_(ω) and *J*_fast_(ω), respectively. To introduce the desired non-Markovianity, we prevent the environment modes in *J*_slow_(ω) from directly influencing the system dynamics. The spectral density separation is formally achieved by defining

12 Jslow(ω)=S(ω,ω*)J(ω)Jfast(ω)=[1−S(ω,ω*)]J(ω)

where *S*(ω, ω*) is the splitting function given by

13 $$S\left(\omega ,\omega^{*}\right)=\{\begin{array}{ll} {\left[1-{\left(\omega /\omega^{*}\right)}^{2}\right]}^{2}, & \omega <\omega^{*}\\ 0, & \omega \geq \omega^{*} \end{array}$$

where ω* is the cutoff frequency.

Next, we treat *J*_slow_(ω) as a source of quasi-static noise that modulates the Bchl’s electronic energies through a Gaussian random process with a standard deviation

14 $$\sigma_{\mathrm{slow}}=\frac{1}{\hbar }\int_{0}^{\infty }J_{\mathrm{slow}}\left(\omega \right)\coth\left(\frac{\beta \hbar \omega }{2}\right)d\omega$$

while *J*_fast_(ω) is explicitly included in the dissipation dynamics in each noise realization as our spectral density. The final result is calculated by averaging the results over many noise realizations.

### Numerical Implementation

The computation of dissipation pathways within the FMO complex requires the partitioning of each chromophore’s spectral density into slow (*J*_slow_(ω)) and fast (*J*_fast_(ω)) components. A uniform cutoff frequency, ω* = 20 cm^–1^, was applied across all Bchls. This specific value was chosen to ensure that less than 5% of the total reorganization energy for each Bchl was attributed to the slow bath component, allowing the majority of the electron-vibrational coupling to be treated explicitly within the simulations. Benchmark HEOM computations (Figure S3 in the SI) show that this choice adequately captures population transfer rates and steady-state populations even for long times >1 ps when the dynamics of some of these slow components of the bath can potentially play a role.

To account for the non-Markovian effects, we performed an ensemble average of over 10,000 independent noise realizations. Importantly, while each realization featured distinct site energies (*E*) drawn from a distribution representing slow-bath component, eq 14, the underlying set of fast spectral density components (*J*_fast_(ω)) remained the same across all realizations. This significantly reduces the computational burden as several crucial quantities, including the line-broadening functions (*g*(*t*)), total reorganization energies (Λ), and the discretized reorganization energies (λ^*j*^ for each *j*-th vibrational mode), needed to be computed only once. Furthermore, to enhance computational efficiency, the calculation of the dissipative potential in eq 7 was vectorized with respect to both the frequency (ω) and time (*t*) grids using the NumPy library in Python. This vectorization allows for the simultaneous evaluation of these quantities for all frequency modes and time points, thereby significantly reducing the overall computation time.

The fast spectral density components, *J*_fast_(ω), were discretized using 4000 effective harmonic oscillator modes equally spaced in frequency. The time integrals required for calculating the population transfer rate constants (eq 2) and the dissipation rates (eq 8) were evaluated numerically by using the trapezoidal rule. This method was applied over a finely spaced time grid with a step size of *Δt* = 0.5 fs and extending to a maximum integration time of *T*_max_ = 30 ps. The trapezoidal rule provides a good balance between accuracy and computational cost for these types of integrals. The propagation of the rate equations governing the electronic populations (eq 1) and the dissipation dynamics (eq 6) was performed by using the fourth-order Runge–Kutta (RK4) method. A consistent time step of 0.5 fs was employed for the RK4 integration, ensuring numerical stability and accuracy throughout the simulation. The accuracy of this approach in generating semiquantitative results for the FMO complex is assessed against the numerically exact HEOM using illustrative models (see Secs. SII–SIII of the Supporting Information). The codes used to obtain the results of this paper are available on GitHub.^93^

## Supplementary Material







## Notes

Details regarding the applicability of the dissipation pathways theory to the Fenna–Matthews–Olson (FMO) complex, the electronic Hamiltonian for the FMO complex, the spectral densities used, the methodology for extracting the vibrational modes, and the dissipation dynamics for a different initial condition (PDF)
Transparent Peer Review report available (PDF)
Set of animations for the extracted vibrational modes around 200 cm^–1^ in each bacteriochlorophyll (ZIP)
