# Supplementary material for: Dissipation Pathways in a Photosynthetic Complex
Source: J Phys Chem Lett. 2025 Dec 12;16(51):13008–16. doi: 10.1021/acs.jpclett.5c02945 (PMC12746460; doi:10.1021/acs.jpclett.5c02945)
Supplement: Supplementary file 1 [file jz5c02945_si_001.pdf]

Supporting Information for Publication

Dissipation Pathways in a Photosynthetic Complex

Ignacio Gustin[a], Chang Woo Kim[b,c], Ignacio Franco[a,d,e]

[a] Department of Chemistry, University of Rochester, Rochester, New York 14627, USA

[b] Department of Chemistry, Chonnam National University, Gwangju 61186, South Korea

[c] The Research Institute for Molecular Science, Chonnam National University, Gwangju 61186, South Korea

[d] Department of Physics and Astronomy, University of Rochester, Rochester, New York 14627, USA

[e] Institute of Optics, University of Rochester, Rochester, New York 14627, USA

E-mail: ignacio.franco@rochester.edu

SI. ELECTRONIC HAMILTONIAN AND SPECTRAL DENSITIES IN THE FMO COMPLEX.

The electronic Hamiltonian has been taken from Adolphs and Renger[1] . All units are in cm^{-1}.

$$\hat{H}_\text{e} = \begin{pmatrix} 410 & -87.7 & 5.5 & -5.9 & 6.7 & -13.7 & -9.9 \\ -87.7 & 530 & 30.8 & 8.2 & 0.7 & 11.8 & 4.3 \\ 5.5 & 30.8 & 210 & -53.5 & -2.2 & -9.6 & 6.0 \\ -5.9 & 8.2 & -53.5 & 320 & -70.7 & -17.0 & -63.3 \\ 6.7 & 0.7 & -2.2 & -70.7 & 480 & 81.1 & -1.3 \\ -13.7 & 11.8 & -9.6 & -17.0 & 81.1 & 630 & 39.7 \\ -9.9 & 4.3 & 6.0 & -63.3 & -1.3 & 39.7 & 440 \end{pmatrix}$$

In turn, the spectral densities have been taken from Ref. [2] and are shown in Fig. SI. All units are in cm^{-1}.

SII. REGIME OF VALIDITY OF THE DISSIPATION PATHWAYS THEORY

In this section, we will test the regime of applicability of our dissipation pathways theory in Fermi's Golden Rule Markovian quantum master equations (QMD-D). To achieve this, we investigate the energy dissipation dynamics of a two-level system interacting with a harmonic thermal environment and compare the results with those obtained from the hierarchical equation of motion (HEOM)[3, 4], a formally exact

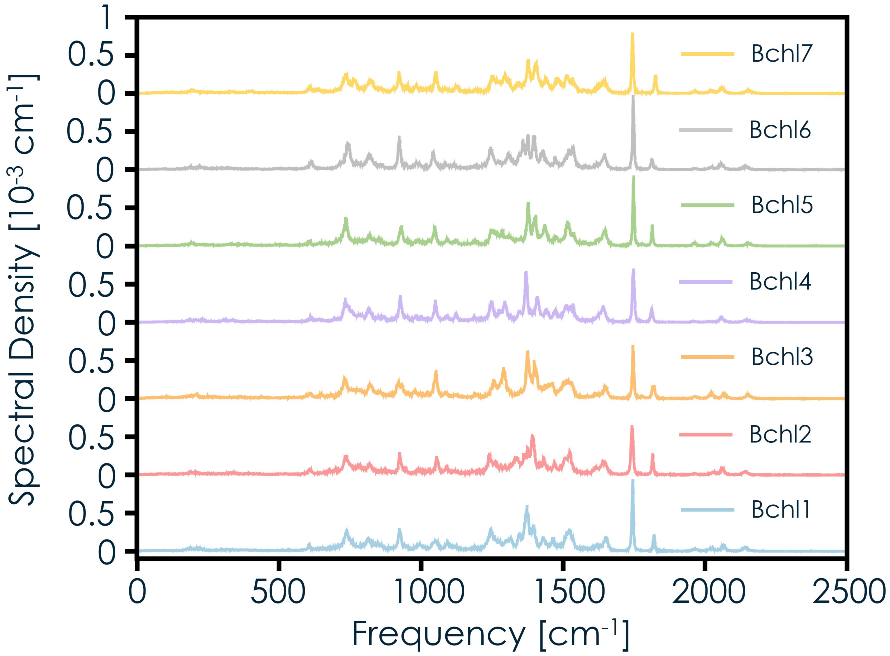

FIG. S1. Spectral densities of the seven Bacteriochlorophyll (Bchl) sites in the FMO complex. Adapted from Kim et al. Phys. Chem. Chem. Phys. 20, 3310 (2018) .[2]

approach. This simplified model was chosen to address the computational limitations associated with dissipation calculations using HEOM via the HEOM-D method.[5]

The HEOM-D method provides a way to extract frequency-resolved energy dissipation into the bath. This is not directly accessible in the standard HEOM framework as the bath is treated collectively and its dynamics is not followed explicitly. The technique works by introducing an additional weakly coupled "probe" harmonic oscillator to the system that is designed to mimic a specific "target" mode of the bath at a given frequency. By calculating the energy dissipated into this probe mode, one can infer the dissipation caused by the actual bath mode at that frequency.

While numerically exact, this technique is computationally demanding. A separate, full HEOM simulation is required for each frequency point of the dissipation spectrum. Furthermore, these calculations face significant convergence challenges in the low-frequency regime. The difficulty arises because the energy gap between a mode's vibrational quantum states is proportional to its frequency ($\hbar\omega$). When this gap is small compared to the available thermal energy (K_BT), many high-energy states become thermally populated. To accurately capture these thermal effects, the simulation must include this large number of populated states, which steeply increases the computational cost.

The interaction between the system and the environment is described by a Drude-Lorentz spectral density $J(\omega) = \frac{2}{\pi}\frac{\lambda\omega^2\omega_c}{\omega^2+\omega_c^2}$, where λ is the reorganization energy and ω_c the cutoff frequency. We chose this functional

form because it can be used to describe low-frequency components in the FMO complex, as shown in the next section. In the simulations, the reorganization energy, λ, was varied across $\lambda = [0.05, 0.2, 1.0, 2.0]$ in Planck atomic units ($h = k_B = 1$), while the energy gap, ΔE, was set to $\Delta E = [1.0, 2.0]$. The temperature was fixed at $T = 1.0$, the electronic coupling at $V = 0.25$ and the cutoff frequency at $\omega_c = 0.5$. To incorporate non-Markovian effects, we implemented the time scale separation method (TSS)[6, 7], which separates the spectral density into slow and fast components, with only the fast component directly influencing the system dynamics while the slow components are treated as a source of slow quasi-static noise that leads to non-Markovian effects. Given that the characteristic time scale of this slow environmental component is significantly longer than that of the dissipation dynamics, its influence is treated as a quasi-static disorder. This effect is then incorporated by averaging over a statistical ensemble of initial conditions (see the details in the Theoretical Methods section of the main paper). The spectral density separation is formally achieved by defining

$$\begin{aligned} J_{\text{slow}}(\omega) &= S(\omega, \omega^*) J(\omega) \\ J_{\text{fast}}(\omega) &= [1 - S(\omega, \omega^*)] J(\omega) \end{aligned} \tag{1}$$

where $S(\omega, \omega^*)$ is the splitting function given by

$$S(\omega, \omega^*) = \begin{cases} [1 - (\omega/\omega^*)^2]^2, & \omega < \omega^* \\ 0, & \omega \geq \omega^* \end{cases} \tag{2}$$

and ω^* is the cutoff frequency which was set to $\omega^* = 0.2$. Averages were taken over 10^3 noise trajectories. For the case with $\lambda = 0.05$, the number of trajectories was increased to 10^4 to ensure numerical convergence.

Fig S1 shows the cumulative dissipation using HEOM-D (red lines) and our approach based on the Fermi's Golden Rule quantum master equation with time scale separation, QMD-D-TSS (black dashed lines). The excited state population is initially placed at either the lower-energy site (Fig. S2a) or the higher-energy site (Fig. S2b). The results show that dissipation profiles obtained from QMD-D-TSS closely match those from HEOM-D across all parameter regimes, with increasing accuracy as the energy gap increases or the reorganization energy decreases, approaching the regime of applicability for the QMD, which is valid in the weak-coupling limit where the electronic coupling strength is small relative to the energy gap between system states. Due to computational limitations, HEOM-D has difficulty resolving dissipation for frequencies below $\omega = 0.2$, while our method remains efficient. These findings confirm that QMD-D-TSS reliably captures key features of dissipative dynamics observed in HEOM-D simulations. Additionally, our method can be straightforwardly extended to more complex systems, including those with highly structured spectral densities, as long as we remain within the QMD's regime of applicability. As shown below, this is the case in the Fenna-Matthews-Olson (FMO) complex.

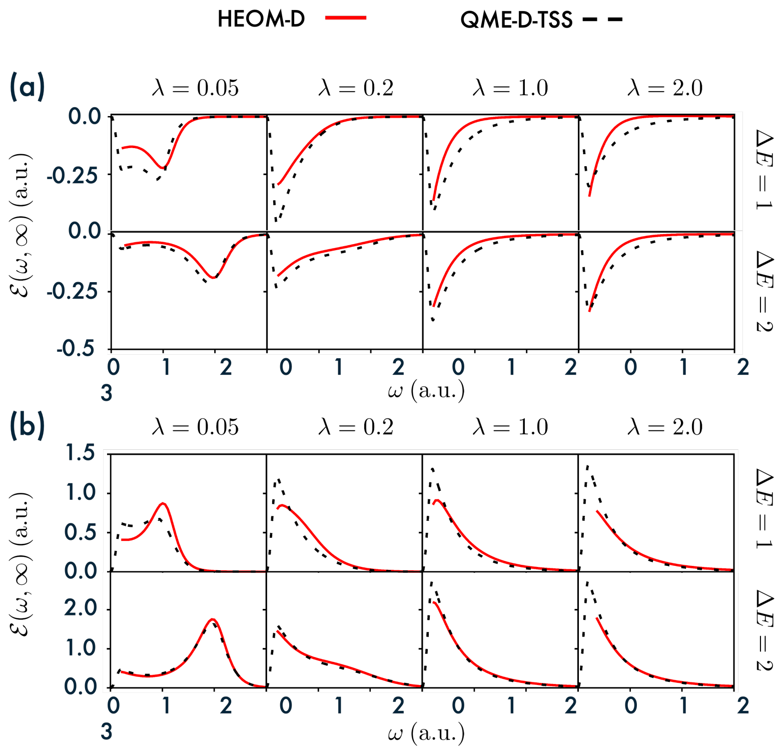

FIG. S2. **Steady-state cumulative dissipation $\mathcal{E}(\omega, \infty)$ in a 2 level system using both QMD-D-TSS and HEOM-D.** Panel (a) and (b) show the total accumulated dissipation for different reorganization energies (λ) and energy gap (ΔE) when the initial population is in the lowest and highest site energy, respectively. Results from QMD-D-TSS are shown as black dashed lines, while those from HEOM-D are shown as red solid lines. Note that negative values of the dissipation indicate energy absorption.

SIII. APPLICABILITY TO THE FMO COMPLEX

Here, we investigate the applicability of the Markovian quantum master equations to accurately capture population dynamics in the Fenna-Matthews-Olson (FMO) complex.[8] Prior studies[9] have demonstrated that at room temperature, particularly when considering low-frequency vibrations, the QMD agrees with the HEOM.[3, 4] This is the case as QMD effectively mirrors the dynamics captured by HEOM, including transfer rates, steady-state populations, and the timescales over which these states are reached. To explore a more realistic regime, we assign different Drude-Lorentz spectral densities to each site in the FMO complex, see Table S1, whose parameters are chosen to reproduce the low-frequency component of the spectral densities extracted from QM/MM simulations.[2] In turn, the electronic Hamiltonian, as seen in Sec. SI, is taken from Adolphs and Renger[1], as it accurately reproduces the optical spectra of the FMO complex.

Fig. S3 compares the FMO population dynamics at 298 K obtained with HEOM (full lines) and that of the QMD with time scale separation (dashed). Here we set $\omega^* = 20$ cm^{-1} for all spectral densities, and average

the results over 10^4 trajectories. The excitation is placed in Bchl1, the site closest to the chlorosome. Examining the early-time population dynamics in Fig. S3a, we observe some discrepancies in the early-time dynamics of bacteriochlorophyll (Bchl) 1 and bacteriochlorophyll 2, as the QMD cannot capture the electronic coherences as HEOM. However, the agreement is remarkable overall, as it accurately captures the transfer rates and steady-state populations as observed in Fig. S3b, which suggests that QMD-D can be used to describe dissipation dynamics in the FMO complex.

The HEOM simulations employed a hierarchy depth of 5 to balance numerical cost and accuracy, utilizing the perturbative low-temperature correction scheme[10] with Matsubara spectrum decomposition and 10 correction terms.

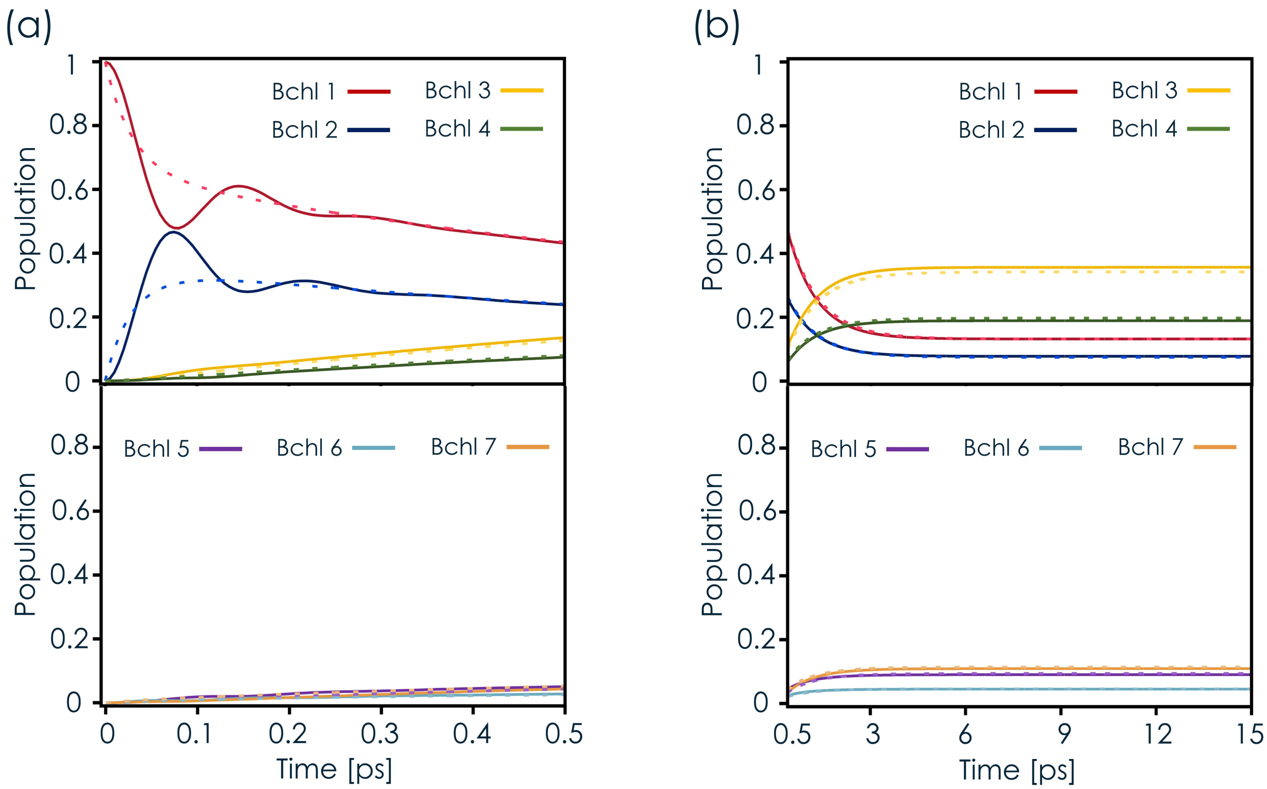

FIG. S3. **Population dynamics in the FMO complex using HEOM (full lines) and the quantum master equation with time scale separation (QMD-TSS, dashed lines).** Panel (a) and (b) correspond to the early and long-time dynamics, respectively. The initial excitation is at Bchl1.

TABLE S1. Spectral density parameters for low-frequency component in the FMO complex. Each spectral density is modeled as a Drude-Lorentz type, $J(\omega) = \frac{2}{\pi} \frac{\lambda \omega^2 \omega_c}{\omega^2 + \omega_c^2}$, where λ is the reorganization energy and ω_c the cutoff frequency.

Site	Reorganization energy λ [cm^{-1}]	cutoff frequency ω_c [cm^{-1}]
Bchl1	40	140
Bchl2	40	140
Bchl3	55	120
Bchl4	20	160
Bchl5	20	110
Bchl6	27	110
Bchl7	32	132

SIV. EXTRACTION OF THE VIBRATIONAL MODES PREDOMINANTLY ACTING IN THE DISSIPATION

The structures of BChl molecules were sampled from the molecular dynamics simulation based on classical mechanics. The molecular force field[11] and the procedure to prepare the simulation box were the same as those used to generate the spectral densities,[2] with the equilibration and subsequent production run lasting for 1 ns and 10 ns, respectively. During the production run, the structures were recorded every 5 fs to preserve dynamical information along the trajectory, resulting in 2×10^6 different conformations for all seven BChl molecules.

Initially, we processed each 100 ns trajectory of the core porphyrin-based moiety of each BChl molecule (see Fig. S4), sampled at 10 fs intervals in valence internal coordinates, $\vec{Z}(t)$. For the definition of the valence internal coordinates, see Table S2 and Fig. S4. Internal coordinates were chosen for their invariance to molecular rotations and translations.

We calculated the average structure, $\vec{Z}_{\text{avg}}$, over the entire trajectory and then determined the structural displacements, $\Delta\vec{Z}(t)$, using the following equation:

$$\Delta\vec{Z}(t) = \vec{Z}(t) - \vec{Z}_{\text{avg}} \tag{3}$$

Subsequently, we defined an initial vector in internal coordinates, $\vec{z}_0$, and calculated its projection amplitude, $A(t)$, throughout the trajectory

$$A(t) = \vec{z}_0 \cdot \Delta\vec{Z}(t). \tag{4}$$

We then conducted a spectral analysis on $A(t)$ to identify the contribution of each frequency component.

TABLE S2. Definitions of the adopted Z-matrix. The corresponding labels of the atomic indices are listed in Fig. S4.

**Bonds**		**Angles**		**Dihedrals**	
2-1	3-2	3-2-1	4-7-10	4-7-10-2	5-4-7-3
4-7	5-4	5-4-7	7-10-2	6-4-7-3	7-10-2-1
6-4	7-10	6-4-7	9-7-10	8-7-10-4	9-7-10-4
8-7	9-7	8-7-10	11-39-34	10-2-1-34	11-39-34-1
10-2	11-39	10-2-1	13-12-1	12-1-2-3	13-12-1-2
12-1	13-12	12-1-2	15-14-13	14-13-12-1	15-14-13-12
14-13	15-14	14-13-12	17-12-1	16-14-13-12	17-12-1-2
16-14	17-12	16-14-13	19-18-16	18-16-17-12	19-18-16-14
18-16	19-18	18-16-17	21-13-12	20-18-16-19	21-13-12-1
20-18	21-13	20-18-16	23-1-12	22-21-10-2	23-1-12-2
22-21	23-1	22-21-10	25-24-23	24-23-1-12	25-24-23-1
24-23	25-24	24-23-1	27-25-24	26-25-24-28	27-25-24-28
26-25	27-25	26-25-24	29-28-25	28-25-24-23	29-28-25-31
28-25	29-28	28-25-24	31-23-1	30-28-25-31	31-23-1-12
30-28	31-23	30-28-25	33-32-17	32-24-23-1	33-32-17-12
32-24	33-32	32-24-23	35-34-1	34-1-23-12	35-34-1-23
34-1	35-34	34-1-23	37-36-35	36-38-39-34	37-36-35-34
36-38	37-36	36-38-39	39-34-1	38-39-34-1	39-34-1-23
38-39	39-34	38-39-34	41-40-38	40-38-39-34	41-40-38-42
40-38	41-40	40-38-39	43-42-11	42-40-38-39	43-42-11-40
42-40	43-42	42-40-38	45-35-34	44-42-11-40	45-35-34-1
44-42	45-35	44-42-11	46-45-31	46-45-31-23	
46-45					

This involved computing the correlation function, $C(t)$, of $A(t)$:

$$C(t) = \langle A(t)A(0)\rangle \tag{5}$$

and applying a Fourier transform to obtain the frequency domain representation, $\tilde{C}(\omega)$:

$$\tilde{C}(\omega) = \text{F.T.}\left[C(t)\right] \tag{6}$$

To focus on optimizing a specific frequency range $[\omega_0, \omega_1]$, while minimizing contributions outside this range, we defined a cost function,

$$f_M = \frac{\mathcal{I}_1 + \mathcal{I}_2}{\mathcal{I}_2} \tag{7}$$

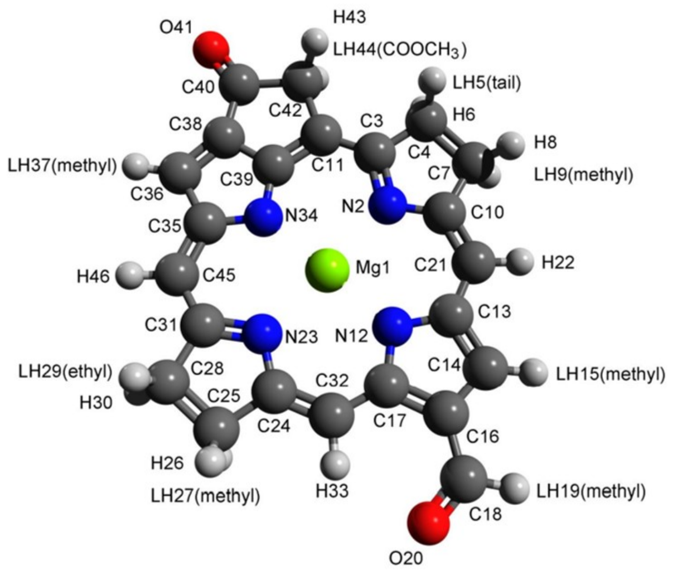

FIG. S4. **Label for atomic indexes.** The atomic indices used to define the valence internal coordinates are listed in Table S2. Hydrogen atoms that serve as links are labeled as "LH," with the corresponding groups they replace indicated in parentheses. Adapted with permission from Ref. [11]. Copyright 2016 American Chemical Society.

where:

$$\mathcal{I}_1 = \int_0^{\omega_0} d\omega \tilde{C}(\omega) + \int_{\omega_1}^{\infty} d\omega \tilde{C}(\omega) \tag{8}$$

$$\mathcal{I}_2 = \int_{\omega_0}^{\omega_1} d\omega \tilde{C}(\omega) \tag{9}$$

Minimizing this cost function involves iteratively refining our initial guess vector, $\vec{z}_0$. We achieve this by first decomposing $\vec{z}_0$ into a linear combination of basis vectors $\{\vec{u}_j\}$, each scaled by a coefficient c_j. Then, to efficiently update these coefficients, we determine the gradient of the cost function with respect to each coefficient:

$$G = \sum_j \frac{\partial f_M}{\partial c_j} \vec{u}_j \tag{10}$$

Here, G is a vector that points in the direction of the steepest ascent in the space defined by the basis vectors $\{\vec{u}_j\}$. We update our guess vector $\vec{z}_0$ by adjusting it in the direction opposite to G (since we are minimizing), scaled by a step size parameter k:

$$\vec{z}_0 = \vec{z}_0 + kG \tag{11}$$

SV. INITIAL CONDITIONS

In the main text, our analysis focused on the physically relevant scenario where the initial excitation is placed on Bchl1, as this is the chromophore closest to the chlorosome antenna complex and the primary entry point for excitation energy. However, Bchl6 is also located near the chlorosome and represents another potential starting point for the energy transfer process. To test the robustness of our conclusions against the choice of initial conditions, we performed an additional simulation initiating the dynamics on Bchl6. While this changes the specific energy transfer pathway through different intermediate chromophores, the core findings of our study remain the same. Figure S5, confirm that energy dissipation is still predominantly driven by low-frequency environmental modes (< 800 cm^{-1}) because their energy is still near-resonant with the electronic energy gaps in the complex. Furthermore, the in-plane breathing modes around 200 cm^{-1} are once again identified as the most significant contributors to the dissipation process and high-frequency vibrations (> 800 cm^{-1}) play a negligible role. This analysis shows that our central conclusions are not an artifact of a specific initial condition but are a general feature of the FMO complex's vibronic structure.

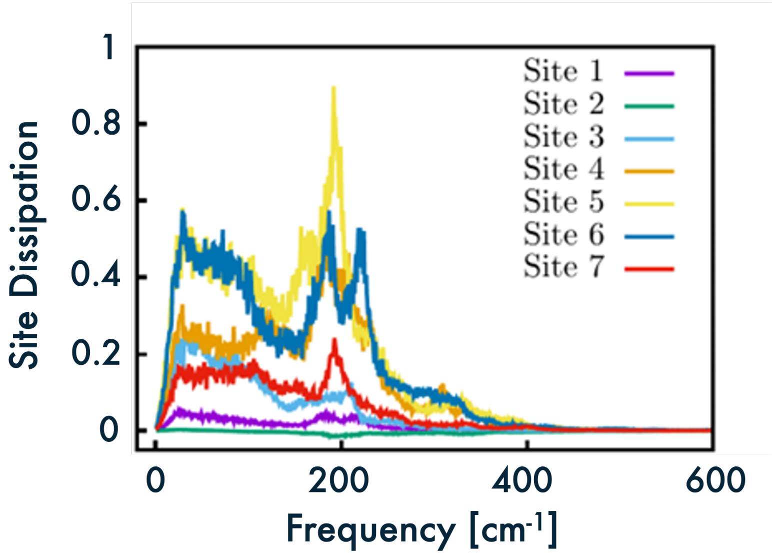

FIG. S5. **Total accumulated site dissipation for initial excitation on Bchl6.** The plot shows the final accumulated energy dissipation as a function of environmental frequency for each of the seven bacteriochlorophyll (Bchl) sites. Although the specific dissipation profiles for each site differ from the case where excitation begins at Bchl1 (see Fig. 1e in the main text), the results confirm that the primary channels for energy exchange remain the same. The process is dominated by low-frequency modes, particularly the in-plane breathing modes around 200 cm^{-1}, which demonstrates the robustness of our core findings.

REFERENCES

[1] J. Adolphs and T. Renger, How proteins trigger excitation energy transfer in the fmo complex of green sulfur bacteria, Biophys. J. **91**, 2778 (2006).

[2] C. W. Kim, B. Choi, and Y. M. Rhee, Excited state energy fluctuations in the fenna–matthews–olson complex from molecular dynamics simulations with interpolated chromophore potentials, Phys. Chem. Chem. Phys. **20**, 3310 (2018).

[3] Y. Tanimura, Numerically “exact” approach to open quantum dynamics: The hierarchical equations of motion (heom), J. Chem. Phys. **153**, 020901 (2020).

[4] T. Ikeda and G. D. Scholes, Generalization of the hierarchical equations of motion theory for efficient calculations with arbitrary correlation functions, J. Chem. Phys. **152**, 204101 (2020).

[5] C. W. Kim, Extracting bath information from open-quantum-system dynamics with the hierarchical equations-of-motion method, Phys.Rev. A **106**, 042223 (2022).

[6] T. C. Berkelbach, T. E. Markland, and D. R. Reichman, Reduced density matrix hybrid approach: Application to electronic energy transfer, J. Chem. Phys. **136** (2012).

[7] A. Montoya-Castillo, T. C. Berkelbach, and D. R. Reichman, Extending the applicability of redfield theories into highly non-markovian regimes, J. Chem. Phys. **143** (2015).

[8] R. Fenna and B. Matthews, Chlorophyll arrangement in a bacteriochlorophyll protein from chlorobium limicola, Nature **258**, 573 (1975).

[9] D. M. Wilkins and N. S. Dattani, Why quantum coherence is not important in the fenna–matthews–olsen complex, J. Chem. Theory Comput. **11**, 3411 (2015).

[10] T. P. Fay, A simple improved low temperature correction for the hierarchical equations of motion, J. Chem. Phys. **157** (2022).

[11] C. W. Kim and Y. M. Rhee, Constructing an interpolated potential energy surface of a large molecule: A case study with bacteriochlorophyll a model in the fenna–matthews–olson complex, J. Chem. Theory Comput. **12**, 5235 (2016).
