# Supplementary material for: Dissipation Pathways in a Photosynthetic Complex
Source: J Phys Chem Lett. 2025 Dec 12;16(51):13008–16. doi: 10.1021/acs.jpclett.5c02945 (PMC12746460; doi:10.1021/acs.jpclett.5c02945)
Supplement: Supplementary file 2 [file jz5c02945_si_002.pdf]

jz-2025-02945x.R1

Name: Peer Review Information for "Dissipation Pathways in a Photosynthetic Complex"

First Round of Reviewer Comments

Reviewer: 1

Comments to the Author

This paper is extremely strong. It is rigorous in its treatment, and perhaps most importantly, it clearly frames a subtle question about how to microscopically engineer and understand energy transfer dynamics. This is crucially important, and this is perhaps the best articulation of the problem that I have seen. This paper should be rapidly published.

As with all models, the model here is approximate, but it is well-implemented and explained. Others will use this as a launching point for new models. This will be an important paper.

If I were to quibble with one word, I don't like the use of "monotonic." The language confuses absorption of the exciton with absorption of vibrational energy. Indeed downhill transfer involves refilling of depopulated vibrational states and energy "borrowing" to facilitate transfer (as well as to perturb the system). I think that this wording could be improved.

Reviewer: 2

Comments to the Author

This Letter, titled “Dissipation Pathways in a Photosynthetic Complex” by G. Ignacio, K. C. Woo, and F. Ignacio, quantifies where electronic excitation energy is dissipated

across pigments and frequencies in Fenna-Matthews-Olson (FMO) complex. Using a time-scale separated framework that treats low-frequency protein modes explicitly (as slow, history bearing) while integrating out high-frequency modes with a second-order FGR kernel, the authors produce site- and frequency-resolved "pathway weights" and time-dependent profiles. The central result is that dissipation is dominated by low-frequency modes (<800 cm^{-1}), with a particularly important ~200 cm^{-1} in-plane BChl mode; high-frequency intramolecular modes contribute negligibly. Early-time non-monotonic flow ("energy uptake" at BChl1/BChl2) is also reported. The method is benchmarked against HEOM on representative models and then applied to FMO complex with structured, pigment-specific spectral densities.

1. Major advance

The Letter delivers a quantitative accounting of dissipation that is simultaneously siteresolved and frequency-resolved. The letter unifies many scattered inferences (low-ω dominance; a ~200 cm^{-1} resonance) into a single computational framework and makes direct predictions about which motions matter on which site in a FMO monomer.

2. Immediate significance

For excitonics in photosynthesis and bio-inspired materials, the work helps shift the conversation from "is the bath Markovian?" to "which parts of the bath do the dissipating, when, and on which pigments?" That's useful for interpreting spectroscopy and for coarse-grained design rules.

A. No Major Concerns

B. Moderate Concerns

1. Improved description of the method

The method used here was recently presented in a series of previous papers that develop a framework for extracting information about dissipation in the bath. The generality of this framework is hard to appreciate in the current paper where the application is focused on a Forster like transport mechanism and the description of the method itself is mostly limited to describing the relevant equations. I encourage the authors to help the audience understand their broader framework they have developed more clearly and, in so doing, help the audience understand how the current work sits in the broader literature.

2. ω* choice and time scale separation consistency

The slow/fast split underpins the approach. Do the authors expect to see the same nonmonotonicity and importance of low frequency mode with a different cutoff frequency. A short schematic explaining what physics migrates between the "slow" and "fast" sectors as ω* moves would help.

3. The role of the initial state and early time

Because the method relies on a Forster-like energy transport mechanism, the initial state is always localized on specific sites and the dynamics are incoherent even from very short time. It would be useful when comparing to the experimental results, particularly those at early time, to comment on the changes to dissipation that arise from both the difference in initial condition (laser pulse excitation) and the coherent dynamics of the exciton (as seen in the beat patterns of the corresponding HEOM calculations in the <200 fs timescale). Specifically, my concern is that when an exciton is delocalized across sites, the corresponding vibrational environments would not relax the same way as when the exciton is entirely on that site.

C. Minor Concerns

4. Clarify Abstract

I would recommend clarifying already at the level of the abstract that these calculations are being done with a second-order perturbation theory so that interested readers have the correct context.

Author's Response to Peer Review Comments:

Reviewer 1: "This paper is extremely strong. It is rigorous in its treatment, and perhaps most importantly, it clearly frames a subtle question about how to microscopically engineer and understand energy transfer dynamics. This is crucially important, and this is perhaps the best articulation of the problem that I have seen. This paper should be rapidly published. As with all models, the model here is approximate, but it is well-implemented and explained. Others will use this as a launching point for new models. This will be an important paper."

Response: We are grateful for the reviewer's very positive and encouraging evaluation of our work. We greatly appreciate the recognition of the rigor of our approach and the importance of clarifying microscopic mechanisms of energy transfer dynamics.

Reviewer 1: "If I were to quibble with one word, I do not like the use of 'monotonic.' The language confuses absorption of the exciton with absorption of vibrational energy. Indeed downhill transfer involves refilling of depopulated vibrational states and energy borrowing to facilitate transfer as well as to perturb the system. I think that this wording could be improved."

Response: We thank the reviewer for their insightful comment regarding the use of the term *non-monotonic*. We agree that our original wording could be misinterpreted as referring to vibrational energy absorption rather than to the exchange of energy between the system and the thermal environment. Our intention was to emphasize that the net energy flow between the excited electronic states (of the bacteriochlorophylls) and their surrounding environment is not unidirectional in time. Specifically, the system transiently absorbs energy from the environment to overcome thermal activation barriers before the net

dissipation process begins. We have revised the text to make this clearer and to avoid potential confusion with other meanings of *non-monotonic*.

Reviewer 2: " This Letter, titled "Dissipation Pathways in a Photosynthetic Complex" by G. Ignacio, K. C. Woo, and F. Ignacio, quantifies where electronic excitation energy is dissipated across pigments and frequencies in Fenna-Matthews-Olson (FMO) complex. Using a timescale separated framework that treats low-frequency protein modes explicitly (as slow, history bearing) while integrating out high-frequency modes with a second-order FGR kernel, the authors produce site- and frequency-resolved "pathway weights" and time-dependent profiles. The central result is that dissipation is dominated by low-frequency modes (<800 cm^{-1}), with a particularly important ~200 cm^{-1} in-plane BChl mode; high-frequency intramolecular modes contribute negligibly. Early-time non-monotonic flow ("energy uptake" at BChl1/BChl2) is also reported. The method is benchmarked against HEOM on representative models and then applied to FMO complex with structured, pigment-specific spectral densities.

1. **Major Advance**: The Letter delivers a quantitative accounting of dissipation that is simultaneously site-resolved and frequency-resolved. The letter unifies many scattered inferences (low-ω dominance; a ~200 cm^{-1} resonance) into a single computational framework and makes direct predictions about which motions matter on which site in a FMO monomer. For excitonics in photosynthesis and bio-inspired materials, the work helps shift the conversation from "is the bath Markovian?" to "which parts of the bath do the dissipating, when, and on which pigments?" That's useful for interpreting spectroscopy and for coarse-grained design rules."
2. **Immediate significance**: For excitonics in photosynthesis and bio-inspired materials, the work helps shift the conversation from "is the bath Markovian?" to "which parts of the bath do the dissipating, when, and on which pigments?" That's useful for interpreting spectroscopy and for coarse-grained design rules."

Response: We sincerely thank the reviewer for these thoughtful and encouraging comments. We greatly appreciate the recognition of our edort to provide a unified computational framework and its potential implications for interpreting spectroscopy and guiding design principles in excitonics.

Reviewer 2: "The method used here was recently presented in a series of previous papers that develop a framework for extracting information about dissipation in the bath. The generality of this framework is hard to appreciate in the current paper where the application

is focused on a Förster-like transport mechanism and the description of the method itself is mostly limited to describing the relevant equations. I encourage the authors to help the audience understand their broader framework they have developed more clearly and, in so doing, help the audience understand how the current work sits in the broader literature."

Response: We thank the reviewer for their constructive suggestions. In response, we have revised the Introduction and Theoretical Methods sections to explicitly emphasize the generality of our dissipation pathways framework. In particular, we now clarify that the method applies to both harmonic and anharmonic environments. This broader scope is now explicitly stated early in the manuscript to better situate the current work within the context of the general theoretical framework developed in our previous studies.

Reviewer 2: "ω* choice and time scale separation consistency. The slow/fast split underpins the approach. Do the authors expect to see the same non-monotonicity and importance of low frequency mode with a diderent cutod frequency. A short schematic explaining what physics migrates between the "slow" and "fast" sectors as ω moves would help."

Response: We thank the reviewer for this insightful comment regarding the role of the cutod frequency ω*. Following the frozen-modes strategy introduced by Montoya-Castillo, Berkelbach, and Reichman (J. Chem. Phys. 143, 194108, 2015), ω* partitions the bath into two sectors: (i) slow modes, which evolve on timescales longer than those of the system and are treated as quasi-static disorder, and (ii) fast modes, which evolve more rapidly and are explicitly included in the dissipation dynamics. As ω* is varied, modes migrate between these two sectors: increasing ω* increases the slow component contributing to energetic disorder, while lowering it increases the number of modes that directly drive relaxation.

In our simulations of the FMO complex, we set ω*=20 cm^{-1}, a choice that ensures that less than 5% of the total reorganization energy of each chromophore is assigned to the slow sector while still producing population dynamics and detailed balance conditions in semiquantitative agreement with numerically exact HEOM benchmarks (see Fig. S3). We agree with the reviewer that a systematic exploration of the dependence on ω* would be an interesting supplementary study. However, given the HEOM benchmark it is beyond the scope of the present work, which focuses on establishing and applying the dissipation pathways framework to the FMO complex.

Reviewer 2: "Because the method relies on a Förster-like energy transport mechanism, the initial state is always localized on specific sites and the dynamics are incoherent even from very short time. It would be useful when comparing to the experimental results, particularly those at early time, to comment on the changes to dissipation that arise from both the diderence in initial condition (laser pulse excitation) and the coherent dynamics of the exciton (as seen in the beat patterns of the corresponding HEOM calculations in the <200 fs timescale). Specifically, my concern is that when an exciton is delocalized across sites, the corresponding vibrational environments would not relax the same way as when the exciton is entirely on that site."

Response: We thank the reviewer for this insightful observation. In our current implementation, we focus on initially localized excitations, which we believe are relevant to the physiological context of natural photosynthesis, where excitons generated via antenna absorption are expected to be site-localized at early times.
We fully agree, however, that ultrafast spectroscopic experiments often involve delocalized initial states due to coherent laser excitation. The vibrational response of the system in such cases may indeed dider, particularly in the sub-200 fs regime where coherent dynamics dominate. While we do not aim to make detailed, quantitative comparisons with those experiments, we reference them to highlight that phenomena like transient uphill energy transfer are observed—suggesting that some dynamical features are robust to the nature of the initial state.

We agree that extending our framework to treat delocalized excitonic initial states and their associated coherent dynamics is an important direction. In fact, we are actively working on generalizing our approach to capture these edects and hope to report on these developments in future work

Reviewer 2: "I would recommend clarifying already at the level of the abstract that these calculations are being done with a second-order perturbation theory so that interested readers have the correct context."

Response: We appreciate the reviewer's suggestion. To improve clarity, we have revised the abstract to state explicitly that our analysis is based on a second-order perturbative treatment of the od-diagonal system couplings.
