# Supplementary figures and images for: Dissipation Pathways in a Photosynthetic Complex
Source: J Phys Chem Lett. 2025 Dec 12;16(51):13008–16. doi: 10.1021/acs.jpclett.5c02945 (PMC12746460; doi:10.1021/acs.jpclett.5c02945)

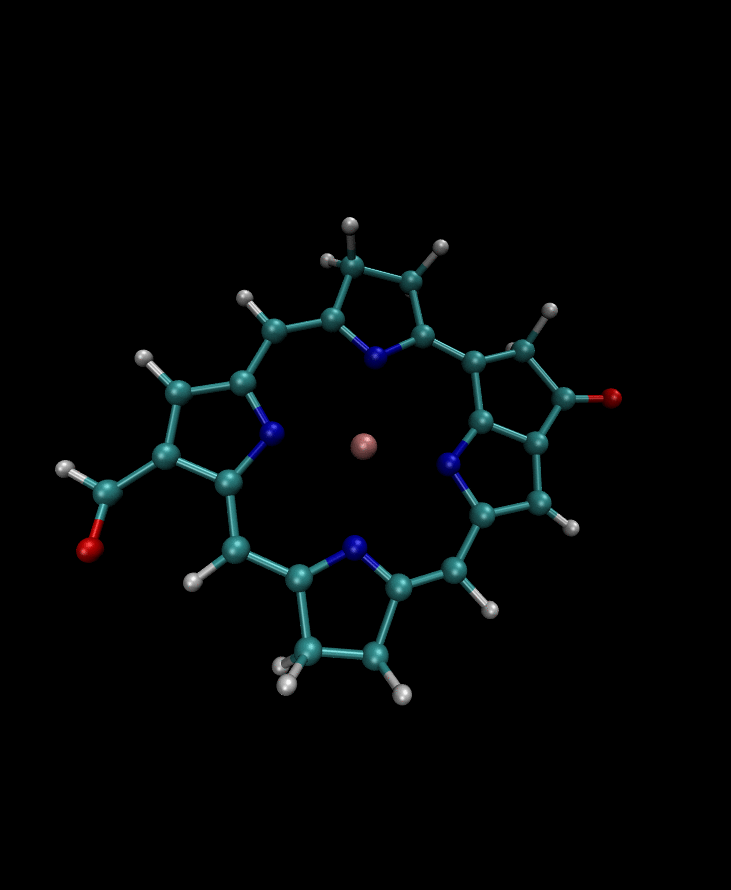

Supplement: Supplementary file 3 [file jz5c02945_si_003.zip › Vibrations-JPCL/BCHL6-Front.gif]

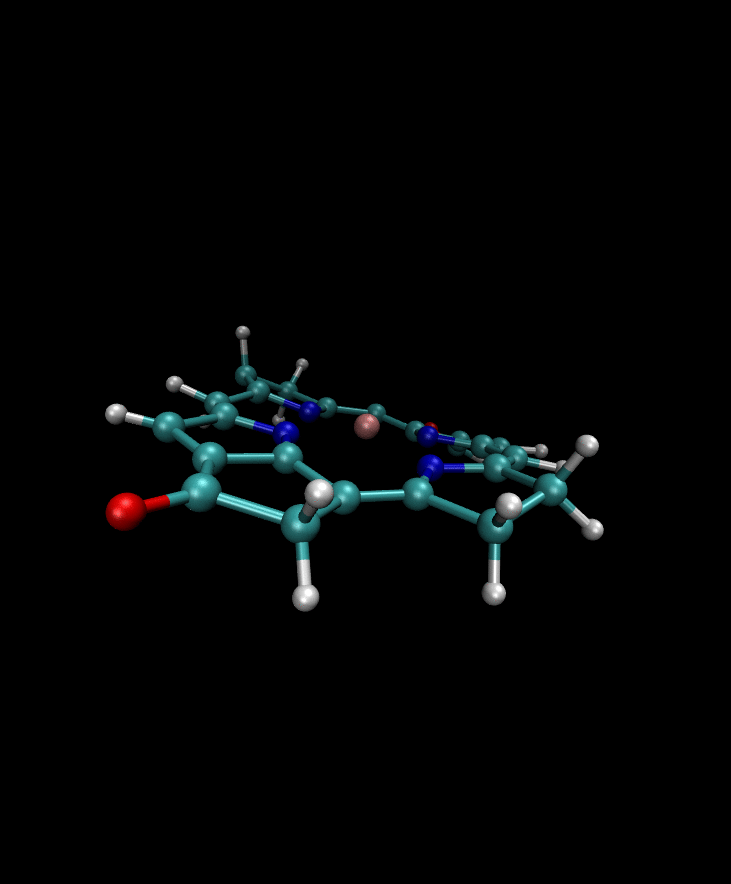

Supplement: Supplementary file 3 [file jz5c02945_si_003.zip › Vibrations-JPCL/BCHL4-Side.gif]

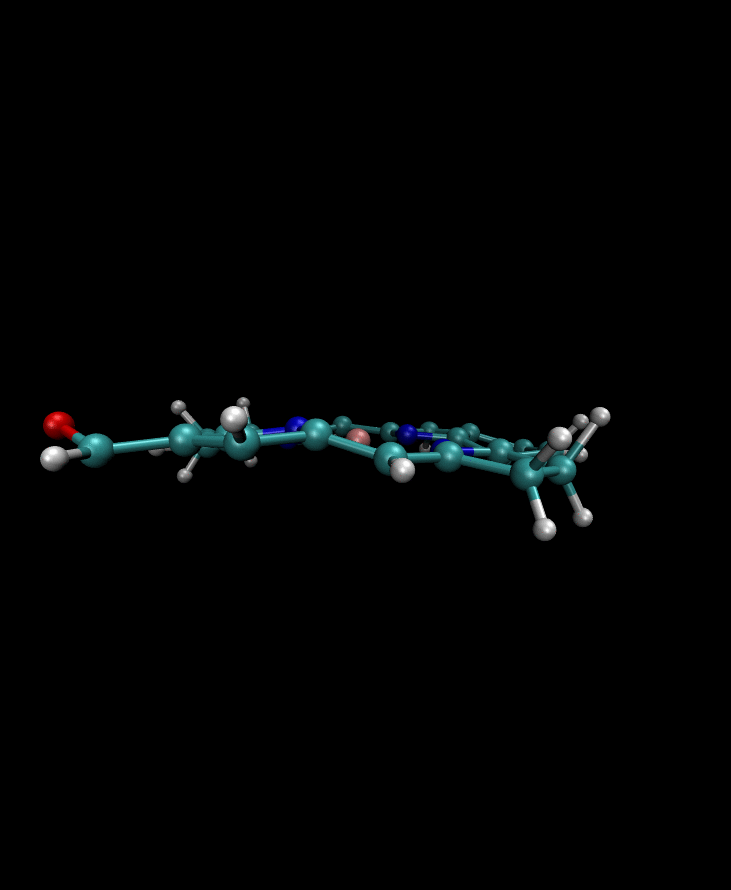

Supplement: Supplementary file 3 [file jz5c02945_si_003.zip › Vibrations-JPCL/BCHL5-Side.gif]

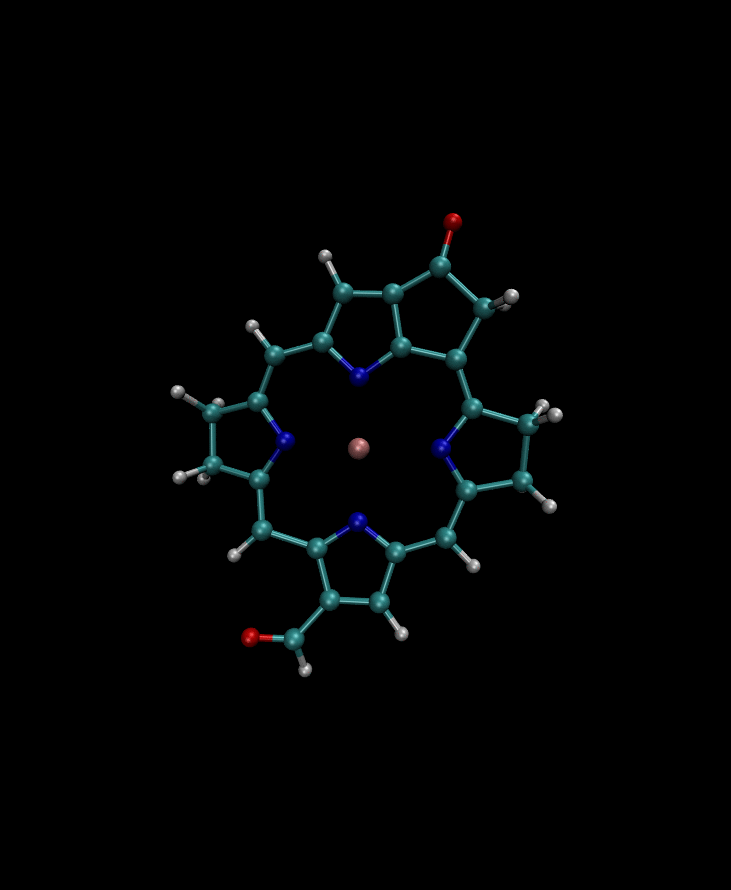

Supplement: Supplementary file 3 [file jz5c02945_si_003.zip › Vibrations-JPCL/BCHL7-Front.gif]

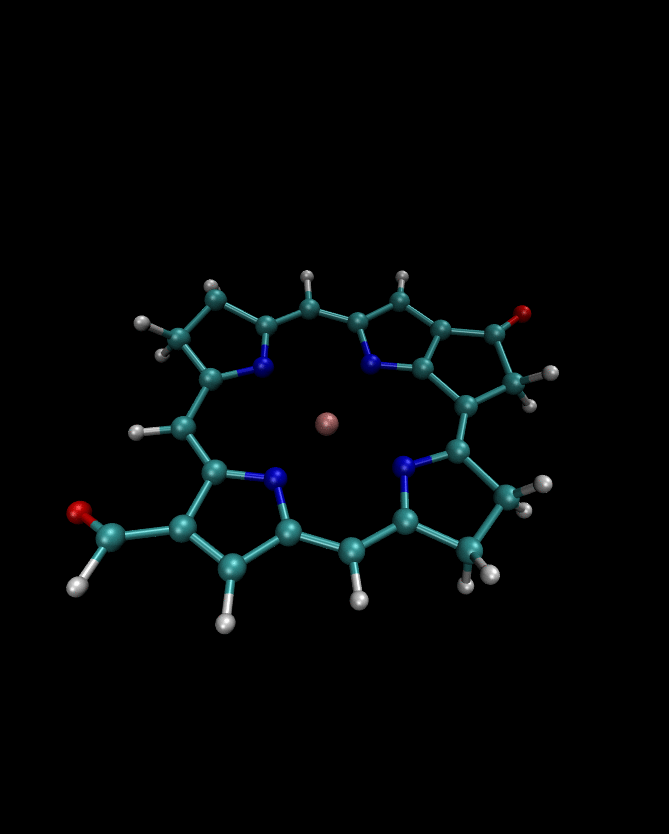

Supplement: Supplementary file 3 [file jz5c02945_si_003.zip › Vibrations-JPCL/BCHL1-Front.gif]

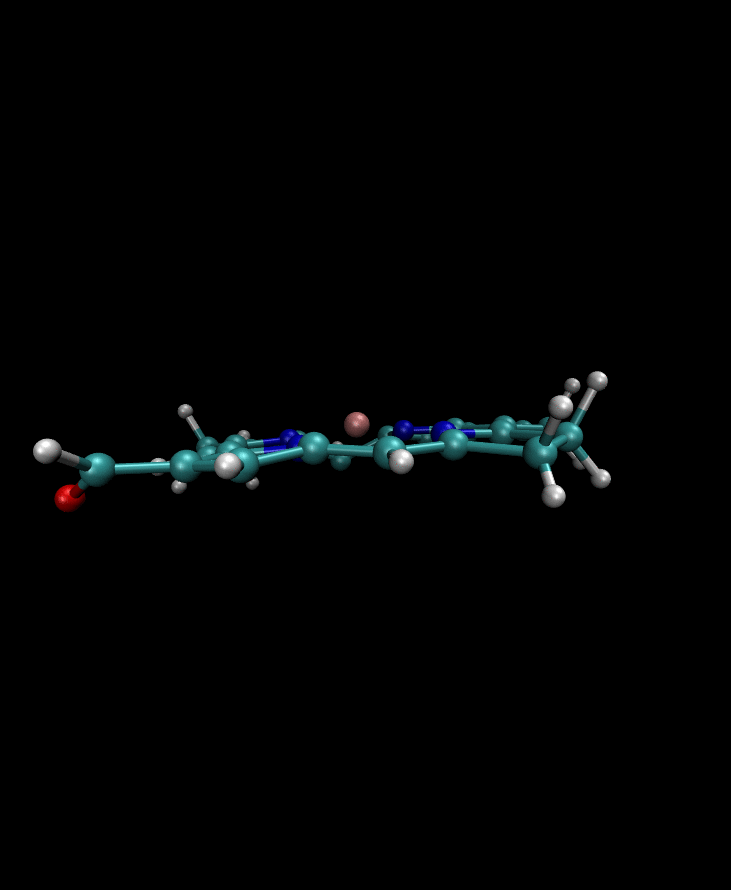

Supplement: Supplementary file 3 [file jz5c02945_si_003.zip › Vibrations-JPCL/BCHL3-Side.gif]

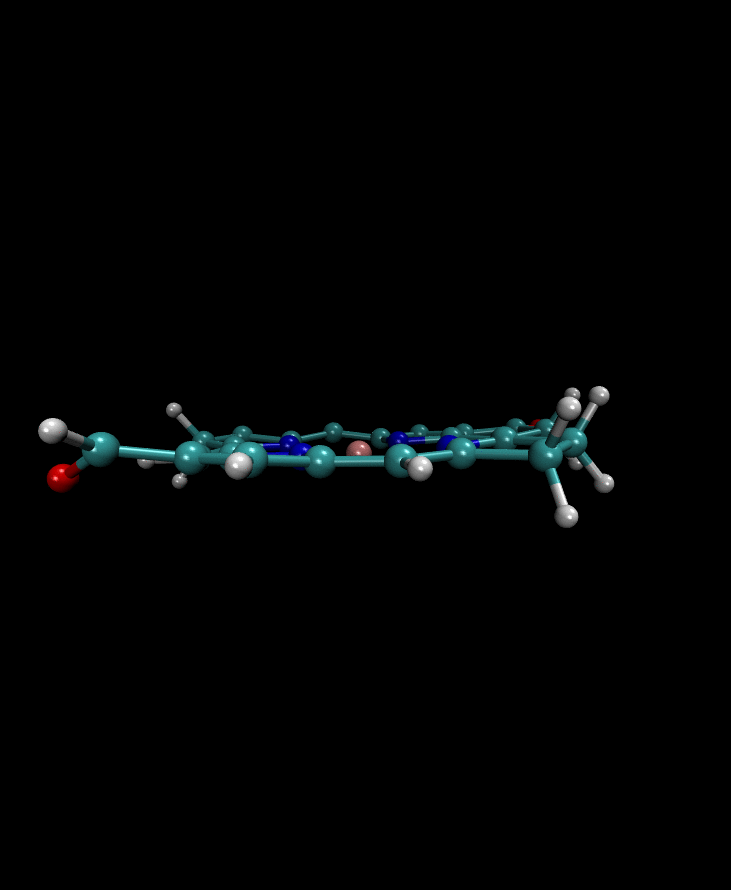

Supplement: Supplementary file 3 [file jz5c02945_si_003.zip › Vibrations-JPCL/BCHL2-Side.gif]

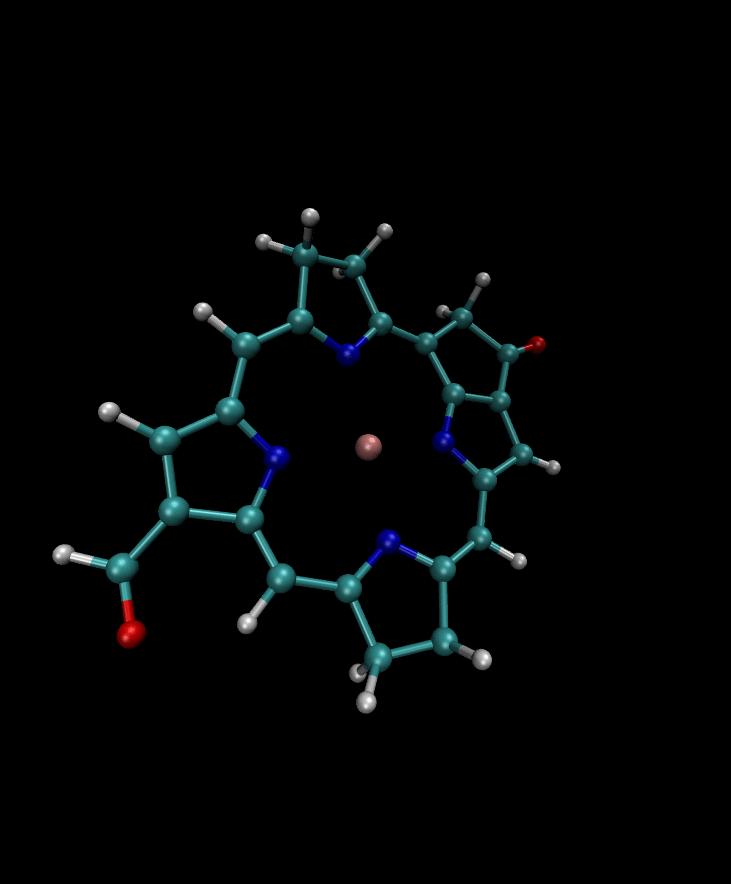

Supplement: Supplementary file 3 [file jz5c02945_si_003.zip › Vibrations-JPCL/BCHL4-Front.gif]

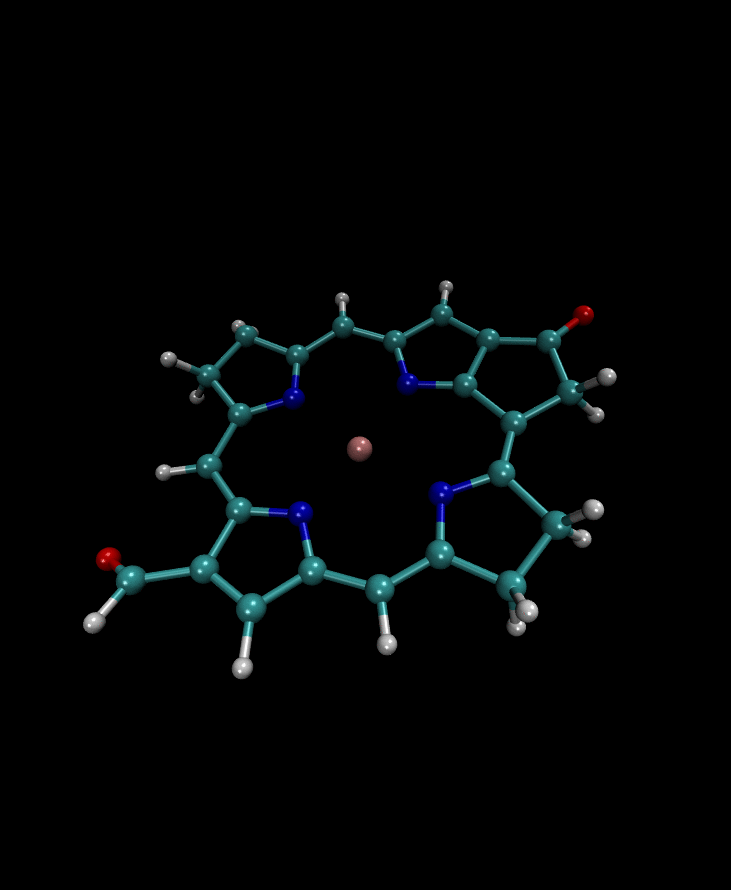

Supplement: Supplementary file 3 [file jz5c02945_si_003.zip › Vibrations-JPCL/BCHL2-Front.gif]

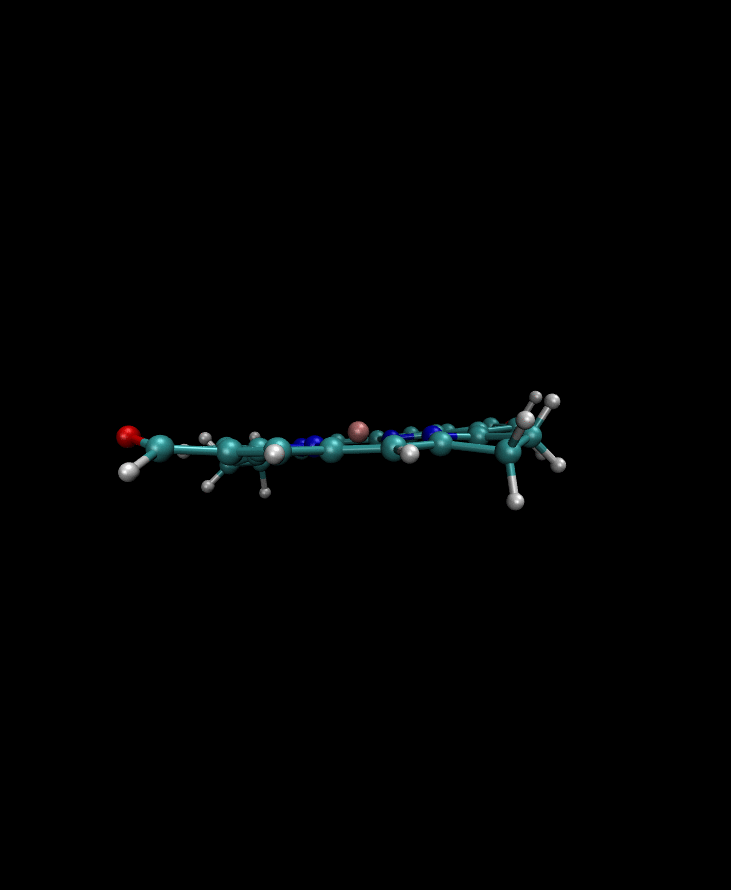

Supplement: Supplementary file 3 [file jz5c02945_si_003.zip › Vibrations-JPCL/BCHL7-Side.gif]

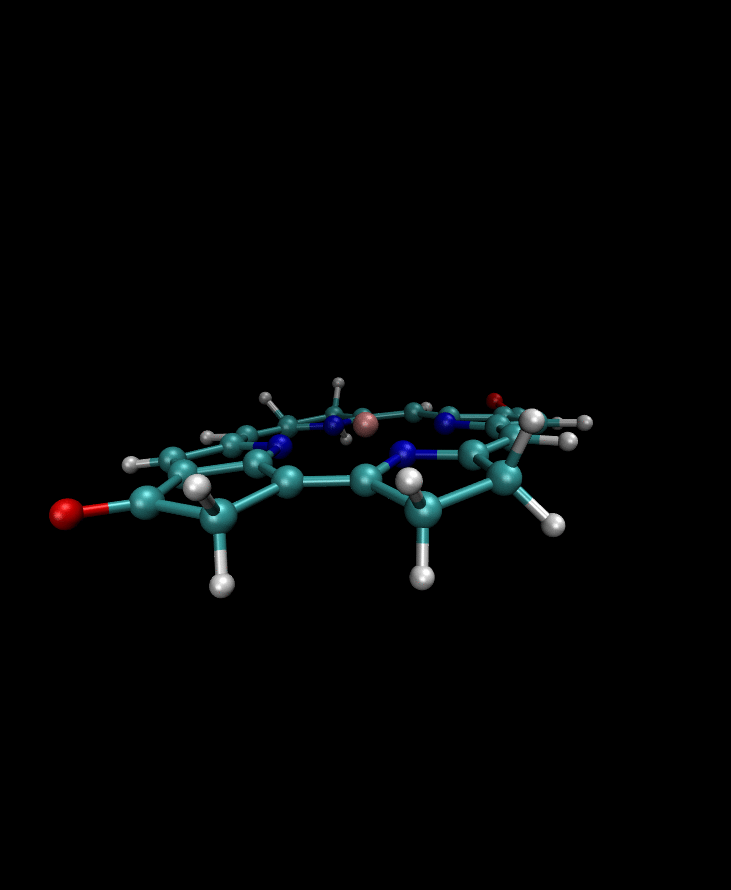

Supplement: Supplementary file 3 [file jz5c02945_si_003.zip › Vibrations-JPCL/BCHL6-Side.gif]

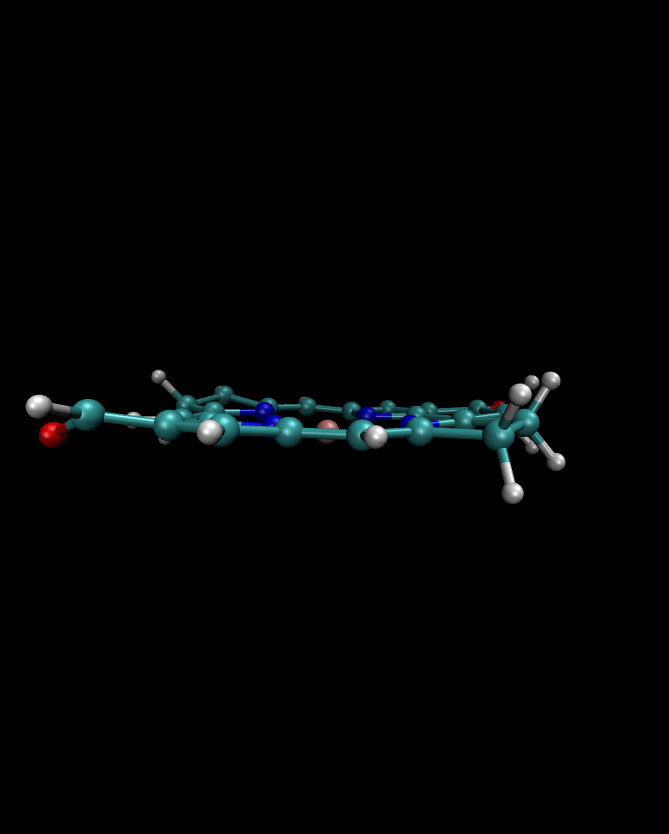

Supplement: Supplementary file 3 [file jz5c02945_si_003.zip › Vibrations-JPCL/BCHL1-Side.gif]

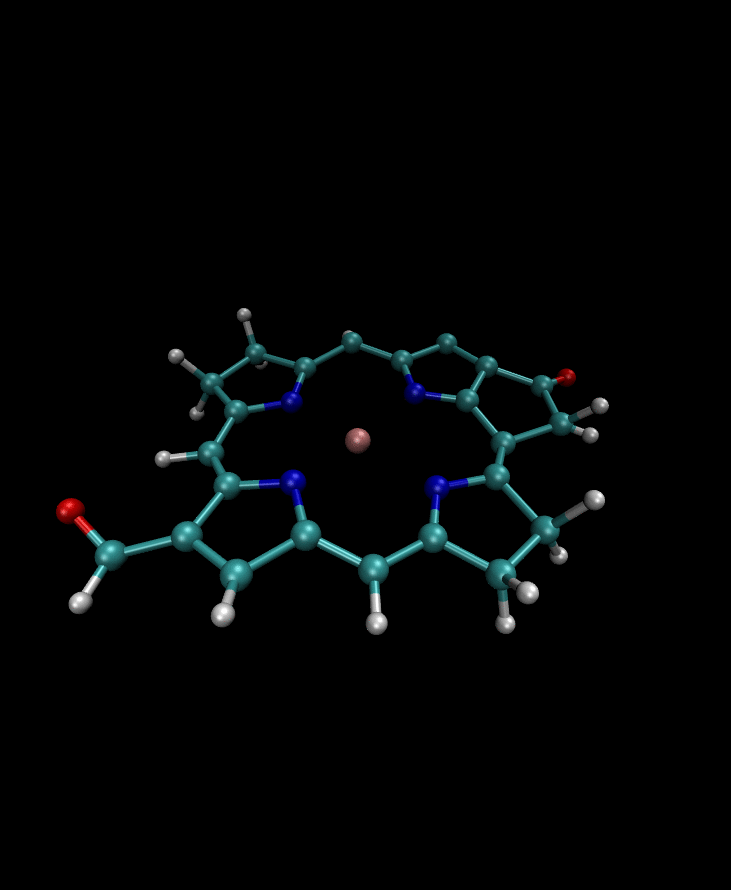

Supplement: Supplementary file 3 [file jz5c02945_si_003.zip › Vibrations-JPCL/BCHL5-Front.gif]

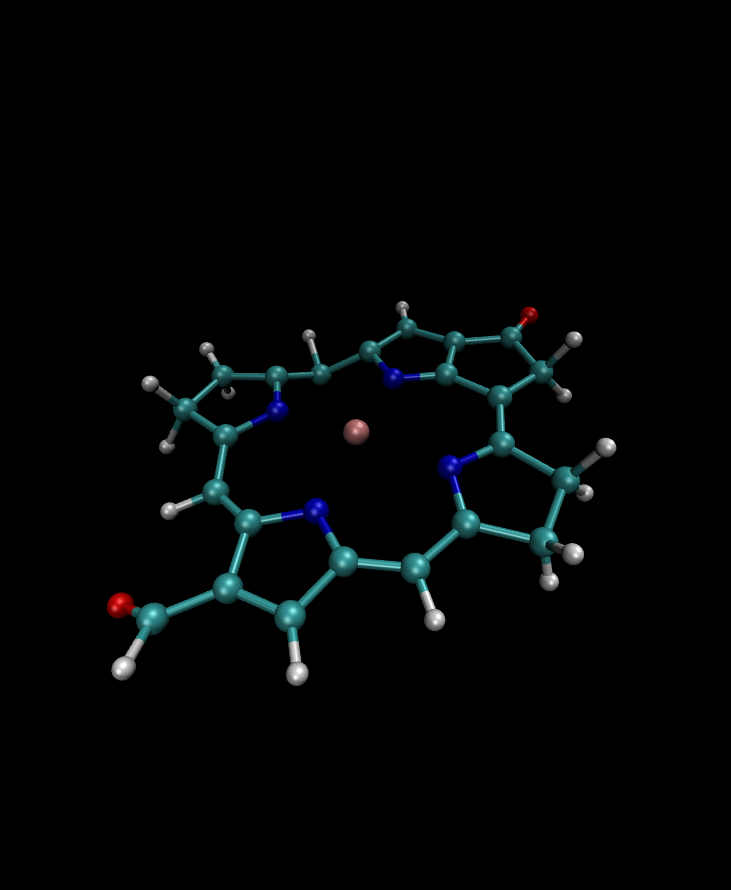

Supplement: Supplementary file 3 [file jz5c02945_si_003.zip › Vibrations-JPCL/BCHL3-Front.gif]
